# Activating mutations in JAK2 and CALR differentially affect intracellular calcium flux in store operated calcium entry

**DOI:** 10.1186/s12964-024-01530-z

**Published:** 2024-03-21

**Authors:** Vikas Bhuria, Tobias Franz, Conny Baldauf, Martin Böttcher, Nicolas Chatain, Steffen Koschmieder, Tim H. Brümmendorf, Dimitrios Mougiakakos, Burkhart Schraven, Sascha Kahlfuß, Thomas Fischer

**Affiliations:** 1 Institute for Molecular and Clinical Immunology, Medical Faculty, Otto-von-Guericke University, Magdeburg, Germany; 2 Health-Campus Immunology, Infectiology, and Inflammation (GC-I3), Medical Center, Otto-von-Guericke University, Magdeburg, Germany; 3 Center for Health and Medical Prevention - CHaMP, Otto-von-Guericke University, Magdeburg, Germany; 4 Department of Hematology and Oncology, Medical Faculty, Otto-von-Guericke University, Magdeburg, Germany; 5 Department of Hematology, Oncology, Hemostaseology and Stem Cell Transplantation, Faculty of Medicine, RWTH Aachen University, Aachen, Germany; 6 Center of Integrated Oncology Aachen Bonn Cologne Düsseldorf (CIO ABCD), Aachen, Germany; 7 Institute of Medical Microbiology and Hospital Hygiene, Medical Faculty, Otto-von-Guericke University, Magdeburg, Germany

**Keywords:** Calcium flux, EPO signaling, TPO signaling, MPNs, JAK2-V617F, CALR mutation

## Abstract

**Background:**

Calcium (Ca^2+^) signaling regulates various vital cellular functions, including integrin activation and cell migration. Store-operated calcium entry (SOCE) via calcium release-activated calcium (CRAC) channels represents a major pathway for Ca^2+^ influx from the extracellular space in multiple cell types. The impact of JAK2-V617F and CALR mutations which are disease initiating in myeloproliferative neoplasms (MPN) on SOCE, calcium flux from the endoplasmic reticulum (ER) to the cytosol, and related key signaling pathways in the presence or absence of erythropoietin (EPO) or thrombopoietin (TPO) is poorly understood. Thus, this study aimed to elucidate the effects of these mutations on the aforementioned calcium dynamics, in cellular models of MPN.

**Methods:**

Intracellular Ca^2+^ levels were measured over a time frame of 0–1080 s in Fura-2 AM labeled myeloid progenitor 32D cells expressing various mutations (JAK2-WT/EpoR, JAK2-V617F/EpoR; CALR-WT/MPL, CALR-ins5/MPL, and del52/MPL). Basal Ca^2+^ concentrations were assessed from 0–108 s. Subsequently, cells were stimulated with EPO/TPO in Ca^2+^-free Ringer solution, measuring Ca^2+^ levels from 109–594 s (store depletion). Then, 2 mM of Ca^2+^ buffer resembling physiological concentrations was added to induce SOCE, and Ca^2+^ levels were measured from 595–1080 s. Fura-2 AM emission ratios (F340/380) were used to quantify the integrated Ca^2+^ signal. Statistical significance was assessed by unpaired Student's t-test or Mann–Whitney-U-test, one-way or two-way ANOVA followed by Tukey's multiple comparison test.

**Results:**

Following EPO stimulation, the area under the curve (AUC) representing SOCE significantly increased in 32D-JAK2-V617F cells compared to JAK2-WT cells. In TPO-stimulated CALR cells, we observed elevated Ca^2+^ levels during store depletion and SOCE in CALR-WT cells compared to CALR-ins5 and del52 cells. Notably, upon stimulation, key components of the Ca^2+^ signaling pathways, including PLCγ-1 and IP3R, were differentially affected in these cell lines. Hyper-activated PLCγ-1 and IP3R were observed in JAK2-V617F but not in CALR mutated cells. Inhibition of calcium regulatory mechanisms suppressed cellular growth and induced apoptosis in JAK2-V617F cells.

**Conclusions:**

This report highlights the impact of JAK2 and CALR mutations on Ca^2+^ flux (store depletion and SOCE) in response to stimulation with EPO and TPO. The study shows that the JAK2-V617F mutation strongly alters the regulatory mechanism of EpoR/JAK2-dependent intracellular calcium balance, affecting baseline calcium levels, EPO-induced calcium entry, and PLCγ-1 signaling pathways. Our results reveal an important role of calcium flux in the homeostasis of JAK2-V617F positive cells.

**Supplementary Information:**

The online version contains supplementary material available at 10.1186/s12964-024-01530-z.

## Background

Mobilization of calcium (Ca^2+^) from the extracellular space into the cytosol is an essential signaling pathway involved in regulation of gene transcription, cellular functions and cell growth. The major Ca^2+^ influx pathway in electrically non-excitable cells such as in hematopoietic cells is store operated calcium entry (SOCE). A common mechanism to switch on SOCE is activation of phospholipase C (PLC) which then results in generation of inositol trisphosphate (IP3) mediating the release of Ca^2+^ from intracellular stores such as the endoplasmic reticulum (ER). The decrease in the Ca^2+^ concentration within the ER is sensed by specific proteins such as stromal interaction molecule (STIM) 1 and its homologue STIM2, which results in the opening of Ca^2+^ release activated channels (CRAC) in the plasma membrane as nicely reviewed by Hogan and Rao [1]. We have previously shown that in erythropoietin (EPO) stimulated hematopoietic cells, PLCγ-1 is activated via a non-STAT5 dependent mechanism [2]. Here, we investigated EPO and thrombopoietin (TPO) induced SOCE and PLCγ1 activation in hematopoietic cells exhibiting activating mutations in JAK2 and CALR which are disease-initiating events in myeloproliferative neoplasms (MPNs).

MPNs comprise a group of clonal hematological diseases including polycythemia vera (PV), essential thrombocythemia (ET) and primary myelofibrosis (PMF). These diseases are characterized by increased numbers of leukocytes, erythrocytes and/or platelets. Genetic studies have revealed that in MPN patients, the Janus kinase 2 (JAK2) V617F mutation is found in the vast majority of PV patients (95%) and in approximately 50% of ET and PMF patients [3, 4]. Additionally, various mutations in the Calreticulin (CALR) protein, in particular a 52-base pair deletion (CALR-del52) and 5-base pair insertion (CALR-ins5), occur with varying frequency in the different forms of MPN disease [5, 6]. CALR mutations are not found in PV patients, whereas they affect about 25% of ET and PMF patients. The disease patterns of both JAK2- and CALR-mutated MPNs include, above all, enlargement of the spleen (splenomegaly) and the development of systemic inflammation with high serum concentrations of pro-inflammatory cytokines [7–9]. In addition, there is an increased risk of transformation into acute leukemia or secondary myelofibrosis [10], i.e., a clinical syndrome of increased fibrous tissue formation in the bone marrow and spleen and an associated suppression of blood formation. However, the most frequently encountered risk factor for the increased disease burden and the high mortality rate in MPN patients is a greatly increased incidence of thrombosis and bleeding [11]. This can manifest as arterial or venous thromboembolism, and includes stroke and myocardial infarction. Interestingly, however, the tendency to progress to thrombosis is less pronounced in CALR mutant patients than in JAK2-V617F positive patients. Another common somatic mutation in MPN affects the thrombopoietin receptor (*MPL*) and is discussed elsewhere [12, 13].

Mutations in JAK2, CALR, and MPL activate common Janus kinase/signal transducer and activator of transcription signaling (JAK/STAT) pathways [14, 15], which are essential for the regulation of hematopoietic stem and progenitor cells [16]. These mutations confer hypersensitivity to several cytokines thereby promoting cytokine-induced proliferation and drive clonal expansion of hematopoietic progenitor cells [17, 18].

Many studies have demonstrated that CALR mutated cells enable the pathological activation of the thrombopoietin receptor (TpoR), thereby driving proliferation of hematopoietic cells via the JAK/STAT pathway [19–21]. Although somatic mutations in JAK2, CALR, and MPL show mutual exclusion in MPNs, suggesting that they activate common signaling pathways [22, 23]; our current understating of the mechanisms of action of these mutations resulting in cytokine-hypersensitivity is still insufficient.

Activation of STAT5A/B, PI3K/AKT, and MAPK pathways is important for the regulation of cellular proliferation and apoptosis. While STAT5 is required for the JAK2-V617F-induced MPN phenotype in mice [24], activation of PI3K/AKT is involved in the malignant transformation driven by JAK2-V617F. Further, Ca^2+^ is an important intracellular secondary messenger, which regulates diverse essential cellular functions, like activation of integrins, cell migration, exocytosis and many more [25, 26]. A major Ca^2+^ influx pathway in many cell types is SOCE through CRAC channels [1]. Although, phospholipase Cγ1 (PLCγ-1), an important regulator of Ca^2+^ signaling pathways, has been implicated in the formation of early erythropoiesis [27, 28], the role of mutated JAK2 and CALR on SOCE has remained elusive. Moreover, recent studies on the pathology of erythroid disorders have determined that Ca^2+^ ion hemostasis plays an important role in ineffective erythropoiesis and increased hypersensitivity to stress-related factors [29]. In addition, in one of the earlier published studies from our group, we had provided evidence that PLCγ-1 signaling plays an essential role in EPO-dependent erythropoiesis and erythroid maturation [2]. Additionally, extracellular-signal-regulated kinase (ERK)/mitogen-activated protein kinase (MAPK) signaling pathway, which is one of the effector pathways of the small GTP-binding Ras protein has also been seen to be activated upon changes in the intracellular Ca^2+^ levels [30]. However, currently, little is known whether intracellular Ca^2+^ homeostasis is affected by JAK2 and CALR mutation in the presence or absence of cytokines. The purpose of this study was to determine the effects of JAK2-V617F, CALR-del52 and CALR-ins5 mutations on Ca^2+^ flux from the ER into cytosol, on SOCE and on key signaling pathways in the presence or absence of EPO or TPO, respectively. In addition, we investigated basal (without cytokine stimulation) calcium levels in the 32D mutant cell lines.

## Material and methods

### Cell lines and Treatment

Murine myeloid progenitor 32D cells, carrying the prevalent JAK2-V617F mutation or the JAK2-wildtype (WT) as previously described [31], as well as CALR-WT, CALR-ins5 and CALR-del52 mutations as previously described [32], were employed in the study. The JAK2-WT/V617F cell lines were stably transfected with the erythropoietin receptor (EpoR), while the CALR-ins5 and CALR-del52 cells were stably transfected with the thrombopoietin receptor (MPL) [31, 32]. 32D-JAK2-WT cells display cytokine-dependent growth and are sustained in RPMI medium containing 10% FCS and EPO as a growth factor at a concentration of 1 IU/ml. On the other hand, JAK2-VF cells demonstrate cytokine-independent growth but are also kept in medium supplemented with EPO at a concentration of 1 IU/ml for one and a half weeks (prior to experimentation) to replicate similar conditions across both cell lines. Thus, 32D-JAK2-WT and JAK2-V617F cells, with an ectopic EpoR expression, were cultured in EPO (1 IU/ml) (Epoetin alfa HEXAL) containing RPMI medium with 10% FCS for 1.5 weeks, followed by 16 h of starvation in EPO free RPMI medium. The starved cells were further treated with EPO (5 IU/ml) for 25 min at 37 °C. Moreover, 32D-CALR-WT cells ectopically expressing MPL were cultured in RPMI medium containing 10% FCS and 10% WEHI-3 supernatant as a source of interleukin-3 (IL-3), whereas MPL expressing CALR-del52 and CALR-ins5 cells grow cytokine-independent and were cultured only in RPMI medium containing 10% FCS. The cells were subjected to a subsequent period of 3 h of starvation in WEHI-free RPMI medium supplemented with 1% FCS. Subsequently, the cells were exposed to mTPO (mouseTPO) for 15 min at concentrations of 10 ng/ml and 100 ng/ml for the purpose of conducting western blot analysis and measuring Ca^2+^ flux, respectively.

Furthermore, in order to modulate calcium signaling in the 32D-JAK2-V617F cell line, we conducted inhibition experiments targeting PLC and BAPTA, a calcium chelator. For PLC inhibition, the cells were subjected to incubation with a PLC inhibitor (U-73122, ENZO) and an inactive analog of U-73122 (ENZO) at varying concentrations for a duration of 30 min. Subsequently, the cells were rinsed with PBS and exposed to 5 IU of EPO for 5 and 15 min, followed by additional assays.

Additionally, for BAPTA treatment, the cells were incubated with a BAPTA (SelleckChem) for 120 min. After a subsequent PBS wash, the cells were exposed to 5 IU of EPO for 5 and 15 min, and further assays were conducted.

### Intracellular calcium measurement

Cells were labelled with Fura-2 AM (Life Technologies) for 30 min in RPMI medium supplemented with 10% FCS, 1% L-glutamine, and 1% penicillin/streptomycin. Cells were attached for 30 min to 96-well glass-bottom plates (Fisher) that had been precoated with 0.01% poly-L-lysine (w/v) (Sigma-Aldrich) for 30 min. Intracellular Ca^2+^ measurements were performed using a plate reader (Synergy H1). Cells were stimulated with EPO (5 IU/ml) or mTPO (100 ng/ml) in Ca^2+^-free Ringer solution (155 mM NaCl, 4.5 mM KCl, 3 mM MgCl_2_, 10 mM D-glucose, 5 mM Na HEPES), followed by addition of 2 mM Ca^2+^ Ringer solution (final [Ca^2+^] 1 mM) to induce SOCE. Fura-2 emission ratios (F340/380) were acquired at 510 nm following excitation at 340 and 380 nm every 27 s (Supplementary Fig. 1). Total Ca^2+^ measurement time frame was from 0 to 1080 s, whereas basal Ca^2+^ concentration was measured between 0—108 s. At 109 s EPO/TPO stimulation was started. Consequently, after EPO/TPO stimulation the Ca^2+^ measurement was performed from 109—594 s (store depletion) and store-operated calcium entry (SOCE) was measured from 595—1080 s. F340/380 ratios were quantified by analyzing the integrated Ca^2+^ signal (area under the curve, AUC) after EPO/TPO stimulation or re-addition of extracellular Ca^2+^ and by analyzing the peak F340/380 response after EPO/TPO stimulation under 2 mM Ca^2+^ re-addition (normalized to the baseline F340/380 ratio before Ca^2+^ re-addition) using GraphPad Prism 8.0 software. This methodology, as elucidated by Eckstein et al. (2017), Kahlfuss et al. (2020), Emrich et al. (2022), and Pan et al. (2018) [33–36], has been widely recognized as an established model for investigating store-operated calcium entry (SOCE). Biologically, the peak response represents the maximum intracellular calcium concentration and the AUC reflects the intracellular calcium concentration–time profile/kinetics of the intracellular calcium concentration.

### Proliferation assay

Following a period of starvation, cells were distributed into a 96-well plate at a density of 5000 cells per well, with each well containing 200 μl of RPMI medium. The JAK2-WT and JAK2-V617F cells were exposed to EPO at a concentration of 1 IU/ml, while the CALR-WT, CALR-del52, and CALR-ins5 cells were treated with mTPO at a concentration of 10 ng/ml. Subsequently, the cells were incubated at 37 °C in a CO_2_ cell incubator. Proliferation measurements were taken at 0 h, 24 h, and 48 h. Before each measurement time point, 20 μl of [3-(4,5-dimethylthiazol-2-yl)-5-(3-carboxymethoxyphenyl)-2-(4-sulfophenyl)-2H-tetrazolium (MTS) reagent was added to each well, followed by a further incubation of 3 h at 37 °C in the CO_2_ incubator. After 3 h incubation, the absorbance was measured at 490 nm using an absorbance plate reader.

### Immunoblotting

After starvation, JAK2-V617F and WT cells were treated with EPO (5 IU/ml) and CALR-WT, CALR-ins5 and CALR-del52 cells were treated with mTPO (10 ng/ml). Total protein from cell lines was extracted after termination of each experimental time point using radioimmunoprecipitation assay buffer (RIPA) buffer (20 mM Tris, 1 mM EDTA, 150 mM NaCl, 1 mM EGTA, 1% Triton X-100, protease and phosphatase inhibitor (Protease Inhibitor Cocktail Tablets, Roche). The samples were then centrifuged at 13,000 rpm for 25 min at 4 °C and supernatants collected. Total extracted protein was quantified using DC protein assay kit (Biorad, München) according to manufacturer’s instruction and absorbance was measured at 650–750 nm using a microplate reader (Gen5™ Microplate Reader). The lysate (protein) was mixed with sample buffer and loading dye, denatured by boiling, and then separated on a 8% or 12% polyacrylamide mini-gel. The proteins were then transferred onto nitrocellulose membranes, and then blocked with 5% (w/v) nonfat dry milk or BSA prepared in Tris-buffered saline with 0,5% Tween-20 (Neolab) (TBST) for 1 h at room temperature. The membranes were subjected to an overnight incubation with primary antibodies (see Additional file 1) specific to the protein of interest. Following this incubation, the membranes underwent a washing step and were subsequently exposed to secondary HRP-labeled antibodies for 1 h. Afterward, the membranes were thoroughly washed three times, with each wash lasting for 10 min using TBST buffer. The membranes were then subjected to further incubation with a Western chemiluminescent HRP substrate (ECL) solution (Merk Millipore) and subsequently proteins were detected using a western blot detection machine (Peqlab Biotechnologie GmbH).

To enable the re-probing of the membranes for both total protein and loading control protein, the membranes were stripped using a stripping buffer (containing β-Mercaptoethanol) for 12 min at 50 °C with continuous agitation at 350 rpm. Following this, the membranes were washed for a total duration of 45 min, with each wash lasting 15 min, and subsequently re-blocked with 5% BSA. After the blocking step, the same procedure as described earlier was followed to detect the target protein.

For densitometry, the densitometric signal of the targeted protein was normalized to their total protein or Vinculin/GAPDH and expressed as the density ratio using ImageJ tool.

### Apoptosis assay

In a 24-well plate, 2 × 10^5^ cells of 32D-JAK2-V617F were seeded in 2 ml of EPO-free cell culture medium (RPMI) and incubated for 16 h at 37 °C in the CO_2_ cell incubator. The cells were then treated with EPO for 25 min at 37 °C. The treated cells were then transfered into FACS tubes and washed with 1 ml of PBS containing 1% FCS at 1,400 rpm for 5 min. The supernatant was removed, and the cells were washed again with 500 µl of annexin binding buffer at 1,400 rpm for 5 min. The supernatant was once again removed, and the cells were resuspended in the remaining volume. To stain the cells, 4 µl of Annexin V-Alexa Fluor® 647 was added and incubated for 15 min at room temperature without washing. The samples were measured within 45 min of Annexin V-Alexa Fluor® 647 staining. After 15 min of incubation, 200 µl of Annexin-V Binding buffer was added, followed by the addition of 3 µl of Sytox blue (1:10) just before measurement. For the positive control, half of the cells were subjected to heat shock treatment by heating them at 65 °C for 2 min and immediately placing them on ice for 3 min for heat shock treatment. The heat-treated cells were then pooled together with the other half of healthy cells. The staining process for the positive control was identical to that of the experimental samples.

### Measurement of mitochondrial membrane potential (ΔΨm)

After subjecting 32D-JAK2-V617F cells to varying concentrations of BAPTA (10 μM and 20 μM) and EPO (5 IU) as previously described, a series of experimental steps were conducted. Initially, cells were rinsed and resuspended in Ringer solution (0 mM Ca^2+^), followed by exposure to 100 nM TMRE dye for 30 min at a temperature of 37 °C inside a CO_2_ incubator. Subsequently, the TMRE reaction was terminated by applying ice-cold PBS, and the cells were then centrifuged, and subjected to two additional warm PBS washes. In contrast, the positive control group was treated with a concentration of 13.5 μM of carbonyl cyanide-p-trifluoromethoxyphenylhydrazone (FCCP), an ionophore, for 10 min at 37 °C inside the CO_2_ incubator. Finally, the mitochondrial membrane potential (ΔΨ_m_) was evaluated using flow cytometry (Cytek Northern Lights instrument).

### RNA isolation and sequencing

Total RNA isolation was performed following treatment of JAK2-V617F cells with EPO using the RNeasy Mini Kit (Qiagen) in accordance with the manufacturer's protocol. Subsequently, the concentration of RNA was quantified using a NanoDrop spectrophotometer. Azenta Life Sciences (GENEWIZ Germany GmbH) conducted RNA sequencing on the isolated RNA samples. The aforementioned company generated the resultant data.

### Statistical analysis

Graphs were analyzed using GraphPad Prism Version 8.0 (GraphPad Software Inc., La Jolla, CA, USA). Statistical significance was determined using unpaired Student’s t-test or Mann–Whitney-U-test, one or two-way ANOVA by Tukey’s multiple comparison test. If not indicated otherwise, data are represented as mean ± SEM to provide an estimate of variation. P-values below 0.05 was considered significant. Where no *p*-value is indicated, no statistically significant *(ns)* difference was found. * p ≤ *0.05,* **p < 0.01; ***p < 0.001, **** *p* ≤ 0.0001. Flow cytometry data were analyzed with FlowJo software (BD Bioscience, Franklin Lakes, NJ, USA).

## Results

### Activating JAK2-V617F mutation increases intracellular (cytosolic) Ca^2+^ levels and enhances Ca^2+^ flux upon EPO stimulation

**Fig. 1:**
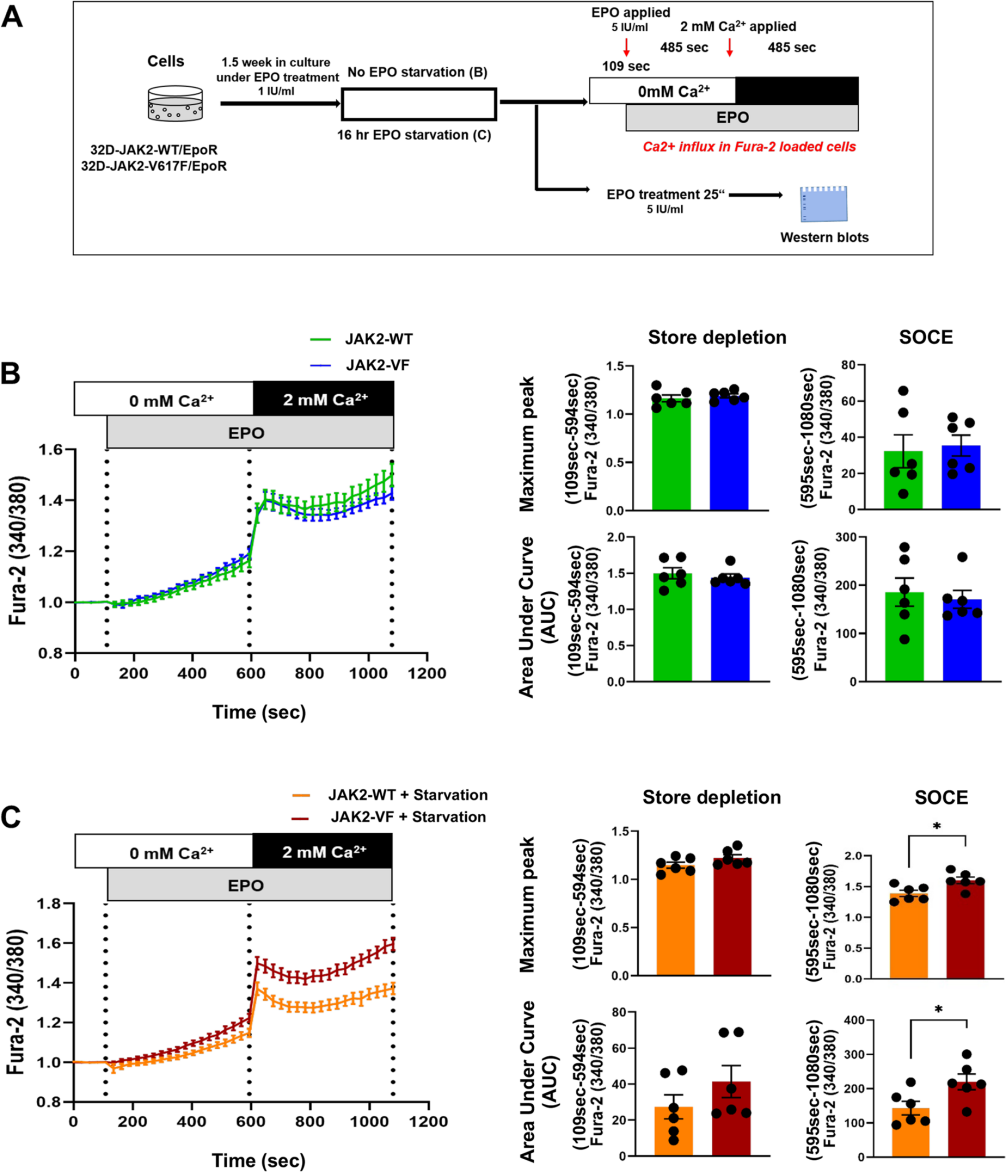
Ectopic expression of JAK2-V617F increases intracellular (cytosolic) Ca^2+^ levels and enhances Ca^2+^ flux upon EPO re-stimulation following cellular starvation. (**A**) Schematic illustration of the experimental design of the study describing the EPO stimulation regimen. (**B**) Ca^2+^ influx in Fura-2 AM loaded 32D-JAK2-WT/EpoR and 32D-JAK2-V617F/EpoR cells under steady state condition. Cells maintained under steady state condition were stimulated with EPO (5 IU/ml) in Ca^2+^-free Ringer solution, followed by addition of 2 mM extracellular Ca^2+^ (left panel). Bar graphs show Ca^2+^ flux response normalized to baseline Ca^2+^ levels (F340/380) as maximum peak and area under the curve (AUC) of store depletion (from 109 to 594 s) and SOCE (from 595 to 1080 s) (right panel). (**C**) Ca^2+^ influx in Fura-2 AM loaded 32D-JAK2-WT/EpoR and 32D-JAK2-V617F/EpoR cells under starved condition. After 16 h of EPO starvation/without starvation, cells were stimulated with EPO (5 IU/ml) in Ca^2+^-free Ringer solution followed by addition of 2-mM extracellular Ca^2+^ (left panel). Bar graphs show Ca^2+^ flux response normalized to baseline Ca.^2+^ levels (F340/380) as quantification of maximal and area under the curve (AUC) of Store depletion (from 109 to 594 s) and SOCE (from 595 to 1080 s) (right panel). Data represents mean ± SEM from 6 independent experiments. Statistical analysis by unpaired Student’s t-test or Mann–Whitney-U-test. * p ≤ *0.05*

32D-JAK2-WT and 32D-JAK2-V617F cells, overexpressing EpoR, were assessed for Ca^2+^ flux and associated key signaling elements in response to EPO stimulation (Fig. 1A). The cell lines, which were under continuous EPO treatment for 1.5 weeks, did not show any differences during ER store depletion after acute EPO stimulation (details on experimental design to measure Ca^2+^ are given in Supplementary Fig. 1), whereas a marginal increase in WT cells was observed during SOCE measurement after treatment with 2 mM calcium (Fig. 1B). Conversely, when the 32D-JAK2-WT and 32D-JAK2-V617F cells were starved and then re-stimulated with EPO, a slight increase in ER store depletion in JAK2-V617F cells was observed (Fig. 1C). Strikingly, SOCE, which was measured as maximum peak and AUC, was significantly increased in JAK2-V617F cells (Max peak: *p* = 0.02; AUC: *p* = 0.03) as compared to JAK2-WT cells (Fig. 1C). Collectively, the slight increase in ER store depletion in JAK2-V617F cells potentiates the extracellular calcium influx through CRAC channels after addition of 2 mM calcium solution.

**Fig. 2:**
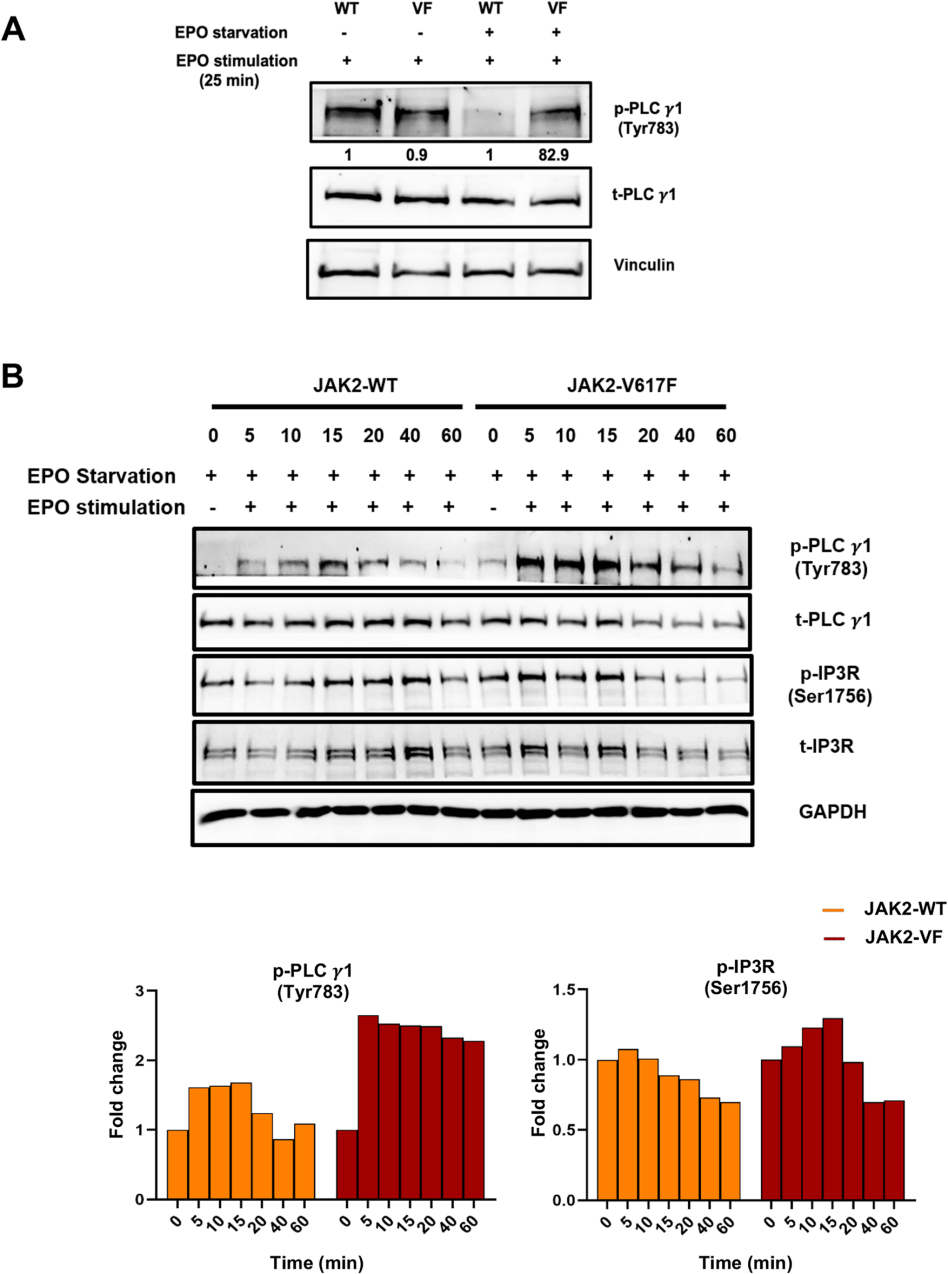
Ectopic expression of JAK2-V617F increases the kinetic sensitivity of PLCγ-1 (Tyr783) phosphorylation and of IP3R (Ser1756) phosphorylation upon EPO stimulation. (**A**) Western blot analysis of target proteins after 25 min of EPO (5 IU/ml) stimulation, following 16 h with or without EPO starvation. Numbers represent values normalized to the respective total protein. (**B**) Western blot analysis of targeted proteins at various times points after EPO (5 IU/ml) stimulation in 32D-JAK2-WT/EpoR and 32D-JAK2-V617F/EpoR cells. Bar diagrams, represent densitometric analysis of western blots, normalized to GAPDH and expressed as a ratio of phospho and total protein

Next, to dissect the underlying molecular mechanisms, we analyzed the downstream and essential elements of the Ca^2+^ signaling pathways. PLCγ-1 and IP3R play important roles in Ca^2+^ signaling regulation. These key elements were evaluated when cells were EPO-stimulated with and without previous EPO starvation. Under nonstarved condition, the PLCγ-1 phospho-protein level (Tyr783) indicating activated PLCγ-1 showed no difference between WT and JAK2-V617F cells when acute EPO stimulation was applied (Fig. 2A). However, when starved cells were re-stimulated with EPO, a 82.9-fold change of p-PLCγ-1 was observed in JAK2-V617F cells (Fig. 2A). We next sought to investigate the kinetics in activation of key signaling nodes upon EPO stimulation. For this, a phospho-protein analysis was performed at different time points in starved cells, starting from 5 min after EPO stimulation up to 60 min (Fig. 2B). Densitometric evaluation revealed that JAK2-V617F cells were hypersensitive towards EPO, as the level of p-PLCγ-1 protein peaked as early as 5 min, whereas in JAK2-WT cells, maximum levels were observed at 15 min post EPO stimulation. Additionally, IP3R was only slightly phosphorylated at Ser1756 in JAK2-WT cells, with a maximum peak at 5 min after acute EPO stimulation, whereas increased levels of p-IP3R were seen in JAK2-V617F cells with a maximum peak at 15 min post EPO stimulation (Fig. 2B, lower panel). Moreover, JAK2-V617F cells exhibited a decelerated decrease in the phosphorylation levels of PLCγ-1 protein (Fig. 2B, lower panel). Further, we conducted an investigation to assess the potential impact of EPO starvation followed by acute EPO treatment on cell death. Our findings revealed that neither the starvation nor the acute EPO treatment demonstrated any significant induction of apoptosis or cell death. Both experimental conditions exhibited only a 7% cell death rate in JAK2-WT cells (Supplementary Fig. 2). Intriguingly, the comparison between WT cells and JAK2-V617F cells demonstrated a notably reduced susceptibility to cell death in the JAK2-V617F cell population, with a mere 1% death rate (Supplementary Fig. 2). In other studies, similar methods of inducing starvation and stimulation have been employed which reinforce the importance of starvation in mitigating the interference arising from endogenous effects of specific cytokines [37].

Collectively, this data provide robust evidence that cells harboring the JAK2-V617F mutation exhibit heightened sensitivity to EPO stimulation in the activation of calcium flux and in activation of signaling pathways involving PLCγ-1 and IP3R. Moreover, our results demonstrate that EPO starvation for 16 h does not significantly induce apoptosis or cell death in JAK2-V617F cells.

### Thrombopoietin-modulated intracellular Ca^2+^ flux in CALR mutated cells

**Fig. 3:**
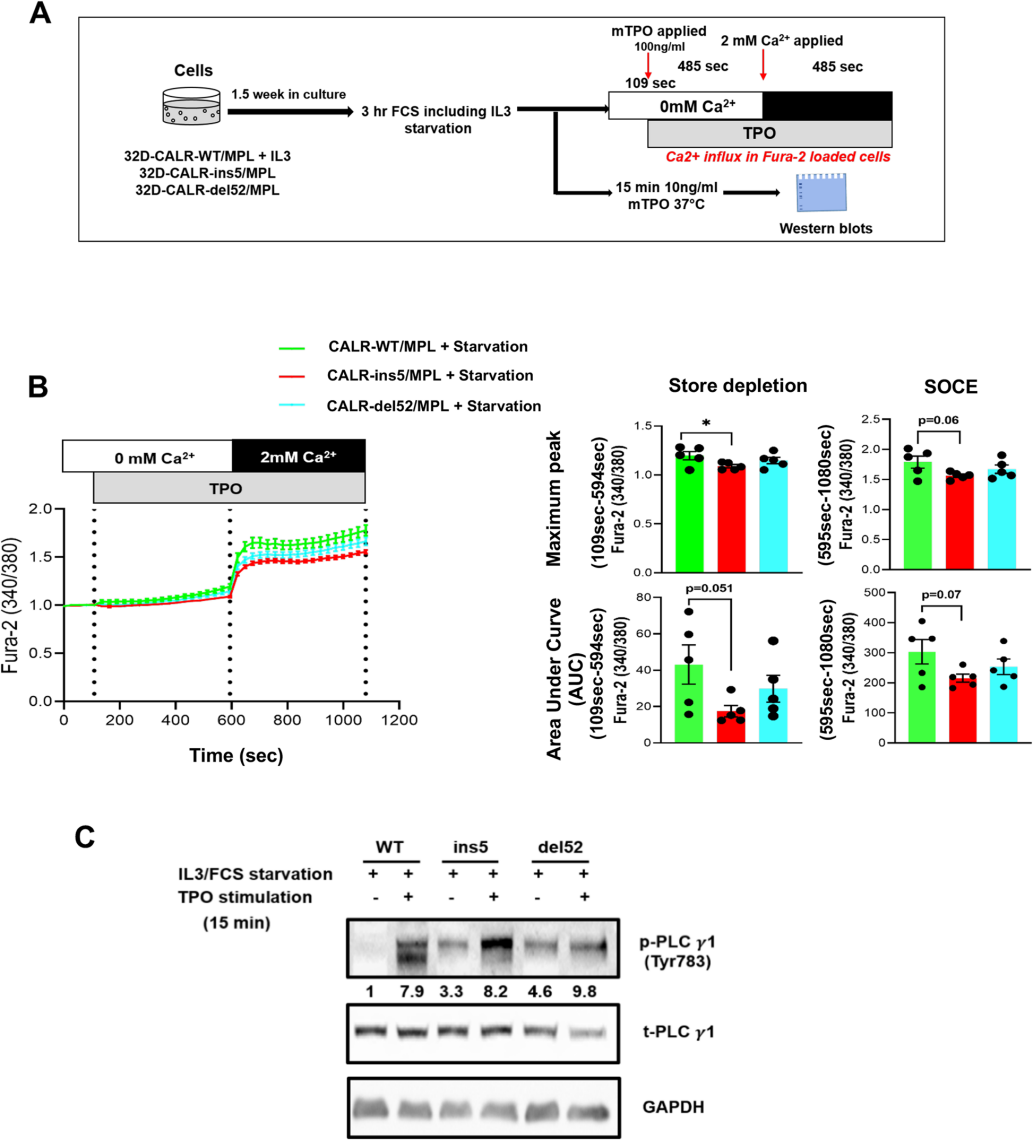
Thrombopoietin (TPO)-modulated intracellular Ca^2+^ flux in CALR mutated cells. (**A**) Schematic illustration of the experimental design of the study describing the TPO stimulation regimen. (**B**) Ca^2+^ influx in Fura-2 AM loaded 32D-CALR-WT/MPL, 32D-CALR-ins5/MPL and 32D-CALR-del52/MPL cells under starvation. After 3 h of IL3/FCS starvation, cells were stimulated with TPO (100 ng/ml) in Ca^2+^-free Ringer solution followed by addition of 2 mM extracellular Ca^2+^ (left panel). Bar graphs show Ca^2+^ flux response normalized to baseline Ca^2+^ levels (F340/380) as maximum peak and area under the curve (AUC) of store depletion (from 109 to 594 s) and SOCE (from 595 to 1080 s) (right panel). Data represents mean ± SEM from 5 independent experiments. Statistical analysis by Mann–Whitney-U-test. * p ≤ *0.05* (**C**) Western blot analysis of target proteins after 15 min of TPO (10 ng/ml) stimulation following 3 h IL3/FCS starvation. Numbers represent values normalized to the respective total protein

CALR mutations were shown to exert their pathophysiological effects by atypical binding of the N-terminal mutated domain of CALR to the extracellular domain of the MPL receptor [38]. Importantly, this mechanism is active in CALR-mutated cells, only [20, 39]. Thus, to investigate the biological effects of CALR mutations in 32D cells, co-transfection with MPL is a prerequisite. Hence, we examined the effects of TPO stimulation on calcium flux in 32D-CALR-WT, 32D-CALR-ins5 and 32D-CALR-del52 cells stably co-expressing MPL. 32D-CALR-WT cells which were cultured in RPMI medium containing 10% FCS and 10% WEHI supernatant as a source of IL-3 were starved from IL3/FCS whereas the IL-3 independently growing 32D-CALR-ins5 and 32D-CALR-del52 cells, were starved from FCS, only (Fig. 3A). Cells were then stimulated with TPO, and Ca^2+^ levels following store depletion and SOCE were measured. The starvation was employed to eliminate the interference caused by the endogenous effects of IL-3 and FCS. This approach allowed for a precise assessment of the influence of TPO stimulation on the activity of intracellular signaling pathways. By removing the confounding variables introduced by IL3 and FCS, the impact of TPO stimulation could be accurately measured. Thus, the experimental design allowed to precisely examine the effects of TPO stimulation when MPL is in an inactive state versus in a constitutively activated state (CALR mutated). This method ensured a clear understanding of the specific effects of TPO on the activation of intracellular signaling pathways. Interestingly, Ca^2+^ concentration was found increased during store depletion (Max peak: *p* = 0.04; AUC: *p* = 0.051) in CALR-WT cells in comparison to CALR-ins5 and del52 cells (Fig. 3B). Further, after applying 2 mM Calcium, the SOCE was also observed to be higher in CALR-WT cells as compared to CALR-ins5 and del52 cells (max. peak: *p* = 0.06; AUC: *p* = 0.07) (Fig. 3B). Western blot analysis of the key components of calcium signaling, PLCγ-1 and IP3R, was also performed to identify the regulatory mechanisms underlying these observed differences.

For p-PLCγ-1, a 7.9- fold change upon TPO stimulation was found in CALR-WT cells, whereas in CALR-ins5 and CALRdel-52 cells, a 2.5-fold and 2.3-fold change, respectively was observed in comparison to the un-stimulated condition (Fig. 3C), thus, given the pivotal role of PLCγ-1 in Ca^2+^ signaling. Western blot analysis confirmed that TPO stimulation had a stronger/higher effect on key regulatory elements of Ca^2+^ signaling in CALR-WT cells compared to the mutant CALR cells.

### Increased baseline Ca^2+^ levels under JAK2-WT and CALR-WT condition

**Fig. 4:**
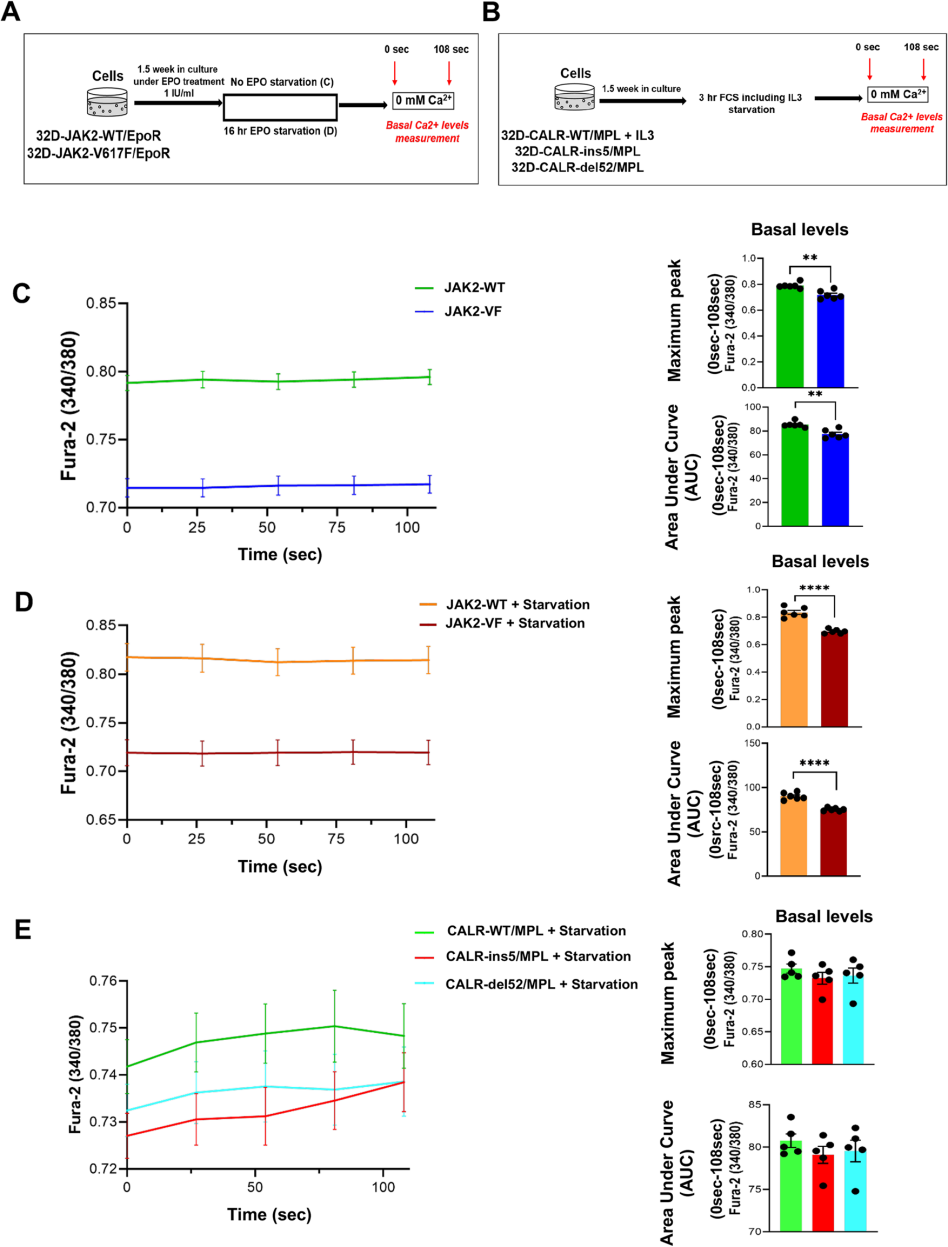
Increased baseline Ca^2+^ levels under JAK2-WT and CALR-WT condition. (**A**) Schematic illustration of the experimental design of the study describing the basal calcium level measurement in 32D-JAK2-WT/EpoR and 32D-JAK2-V617F/EpoR cells under steady state condition and pre-EPO stimulation. (**B**) Schematic illustration of the experimental design of the study describing the basal calcium level measurement in 32D-CALR-WT/MPL and 32D-CALR-ins5/MPL and 32D-CALR-del52/MPL cells pre-TPO stimulation. (**C**) & (**D**) Increased basal Ca^2+^ levels in 32D-JAK2-WT/EpoR cells. Data represents mean ± SEM from 6 independent experiments. Statistical analysis by Mann–Whitney-U-test (for Figure C) and unpaired Student’s t-test (for Figure D). **p < 0.01; **** *p* ≤ 0.0001 (**E**) Increased basal Ca.^2+^ levels in 32D-CALR-WT/MPL cells. Data represents mean ± SEM from 5 independent experiments. Statistical analysis by unpaired Student’s t-test. **p < 0.01; **** *p* ≤ 0.0001

To explore the implication of JAK2-V617F, CALR-ins5 and CALR-del52 mutations on intracellular calcium homeostasis, the 32D-JAK2-WT, 32D-JAK2-V617F, 32D-CALR-WT, 32D-CALR-ins5 and 32D-CALR-del52 cells were investigated under un-starved and starved conditions without acute EPO/TPO stimulation (Fig. 4A, 4B). Unexpectedly, 32D-JAK2-WT cells showed significantly higher cytosolic calcium levels under both un-starved (*p* = 0.001) (Fig. 4C) and EPO-starved (*p* = 0.001) (Fig. 4D) conditions as compared to 32D-JAK2-V617F cells. In CALR cell lines, increased levels of cytosolic calcium were observed in CALR-WT cells in comparison to CALR-ins5 and CALR-del52 cells (Fig. 4E). Collectively, the observation of higher levels of cytosolic calcium in JAK2-WT cells (Fig. 4C, 4D) may result in a reduced inward calcium driving force and impaired SOCE in JAK2-WT cells (Fig. 1C).

### Calcium signaling inhibition prevents EPO induced cell proliferation and induces apoptosis in 32D-JAK2-V617F cells

**Fig. 5:**
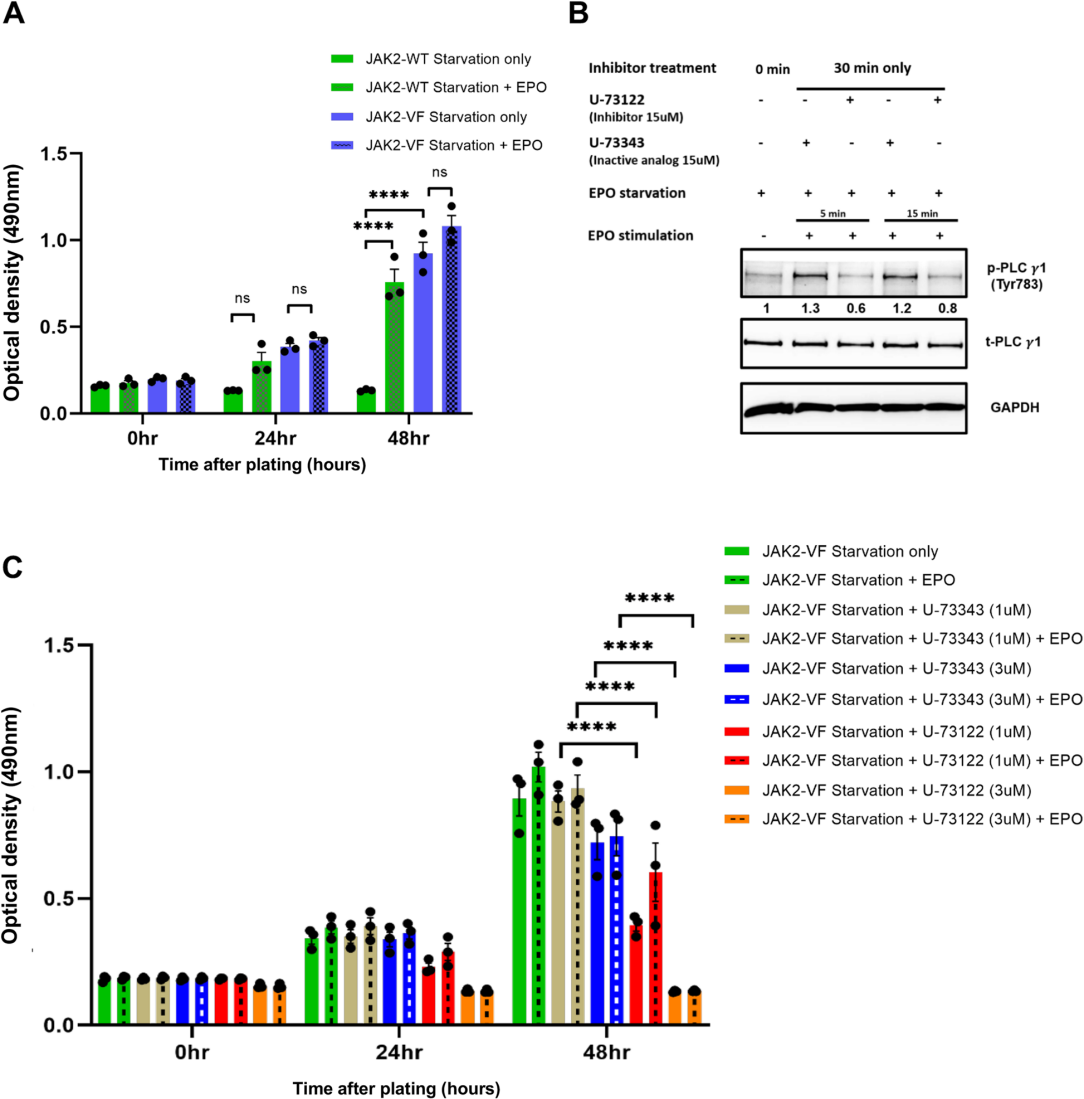
The inhibition of PLCγ-1 effectively inhibits the proliferation of 32D-JAK2-V617F cells induced by EPO. (**A**) Cell proliferation assay of 32D-JAK2-WT/EpoR and 32D-JAK2-V617F/EpoR cells after EPO stimulation (1 IU/ml). Data represents mean ± SEM from 3 independent experiments. Statistical analysis by two-way ANOVA by Tukey’s multiple comparison test. ns *p* > 0.05; **** *p* ≤ 0.0001. (**B**) Western blot analysis of PLCγ-1 after using PLC inhibitor (U-73122) and inactive analog (U-73343) of U-73122 in 32D-JAK2-V617F/EPOR cells. Numbers represent values normalized to the respective total protein. (**C**) Cell proliferation assay of 32D-JAK2-V617F/EpoR cells after concomitant treatment with EPO stimulation (1 IU/ml) and U-73122 or U-73343. Data represents mean ± SEM from 3 independent experiments. Statistical analysis by two-way ANOVA by Tukey’s multiple comparison test. **** *p* ≤ 0.0001

We then investigated the proliferation of JAK2-WT and JAK2-V617F cells with and without EPO stimulation. In accordance with the literature, JAK2-VF cells showed cytokine independent growth upon EPO starvation as compared to JAK2-WT cells (Fig. 5A). Additionally, when the starved cells were stimulated with EPO, a slight increase in proliferation rate was seen in JAK2-VF cells compared to the unstimulated condition (Fig. 5A). Next, we sought to elucidate the impact of EPO-induced Ca^2+^ signaling on the proliferation of 32D-JAK2-V617F cells. Hence, we subjected these cells to treatment with either PLC-specific inhibitor U-73122 or the inactive analog U-73342, and subsequently evaluated their inhibitory impact and proliferative response. As anticipated, brief treatment with U-73122 led to the inhibition of PLCγ-1 phosphorylation (*p* = 0.0001) (Fig. 5B). Remarkably, U-73122 also exhibited a dose-dependent suppression of proliferation of 32D-JAK2-V617F cells (Fig. 5C).

**Fig. 6:**
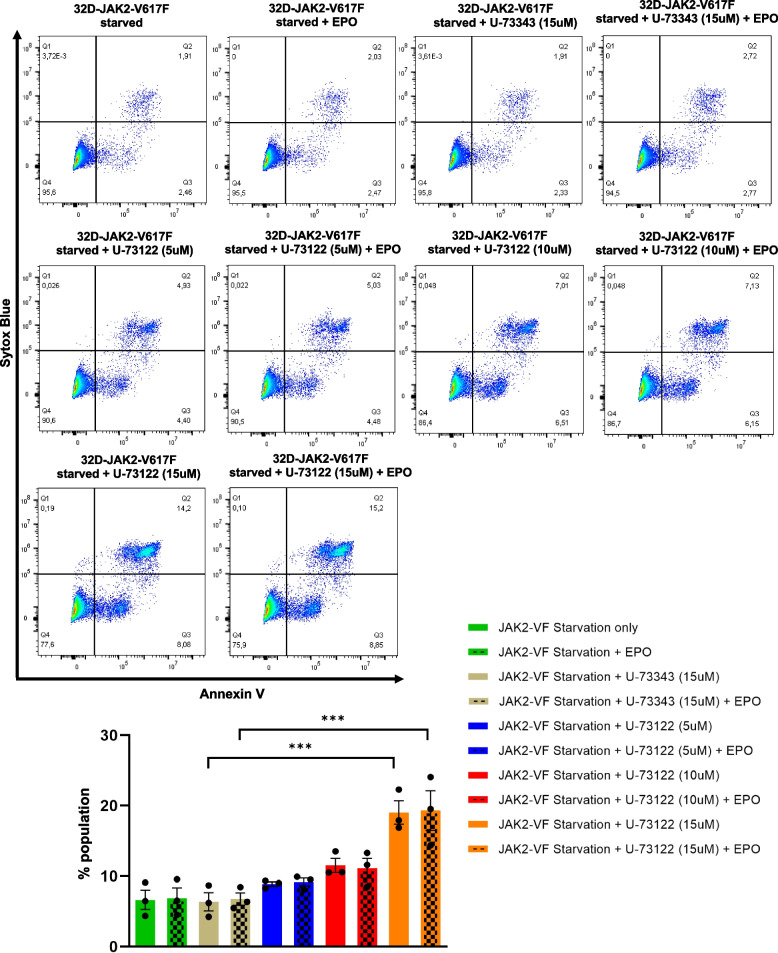
Inhibition of PLCγ-1 triggers apoptosis in 32D-JAK2-V617F cells. Representative flow cytometric dot plots showing the effect of starvation, EPO stimulation and the inhibitory effect of U-73122 or U-73343 on early (right lower quadrant) and late (right upper quadrant) apoptosis in 32D-JAK2-V617F/EpoR cells. Bar data represents mean (sum of early and late apoptosis) ± SEM from 3 independent experiments. Statistical analysis by one-way ANOVA by Tukey’s multiple comparison test. *** p ≤ 0.001

**Fig. 7:**
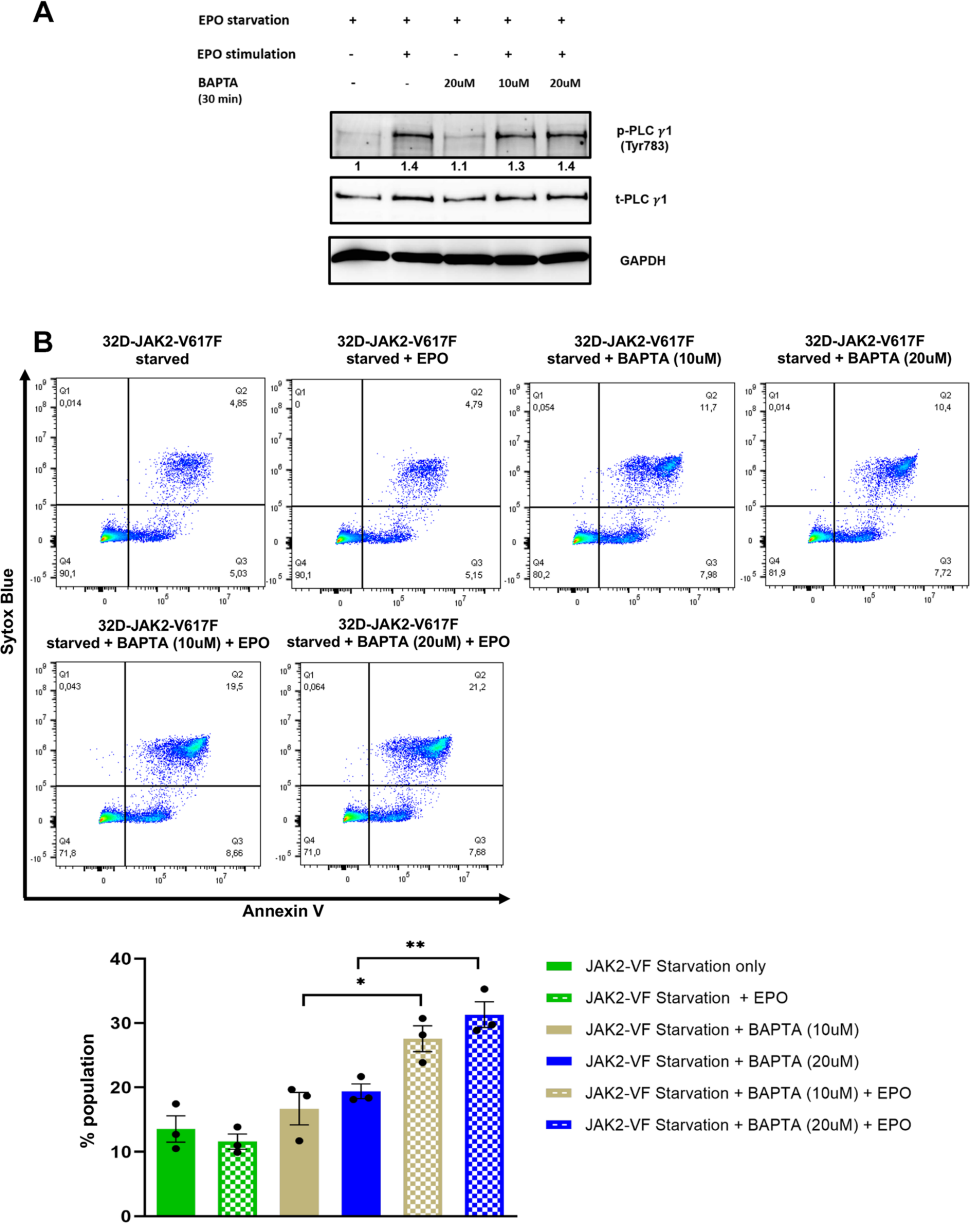
BAPTA treatment induces apoptosis in 32D-JAK2-V617F cells. **A** Western blot analysis of PLCγ-1 after BAPTA treatment in 32D-JAK2-V617F/EPOR cells. Numbers represent values normalized to the respective total protein. **B** Representative flow cytometric dot plots showing the effect of starvation, EPO stimulation and inhibitory effect BAPTA on early (right lower quadrant) and late (right upper quadrant) apoptosis in 32D-JAK2-V617F/EpoR cells. Data represents mean ± SEM from 3 independent experiments. Statistical analysis by one-way ANOVA by Tukey’s multiple comparison test. * *p* ≤ 0.05; ** *p* < 0.01.

Furthermore, we explored the potential consequences of inhibiting Ca^2+^ signaling on cell apoptosis. Our findings demonstrate that the inhibition of PLCγ-1 significantly induced apoptosis in 32D-JAK2-V617F cells (*p* = 0.001) (Fig. 6). To ascertain whether this apoptotic effect was solely attributed to the inhibition of Ca^2+^ signaling, we employed a Ca^2+^-specific chelator, BAPTA. Intriguingly, BAPTA treatment did not show any effect on the phosphorylation of PLCγ-1 (Fig. 7A). However, BAPTA treatment alone induced a marginal increase in apoptosis in these cells (Fig. 7B). Furthermore, when cells treated with BAPTA were subsequently exposed to EPO stimulation, a notable increase in the rate of apoptosis was observed (*p* = 0.01) (Fig. 7B).

**Fig. 8:**
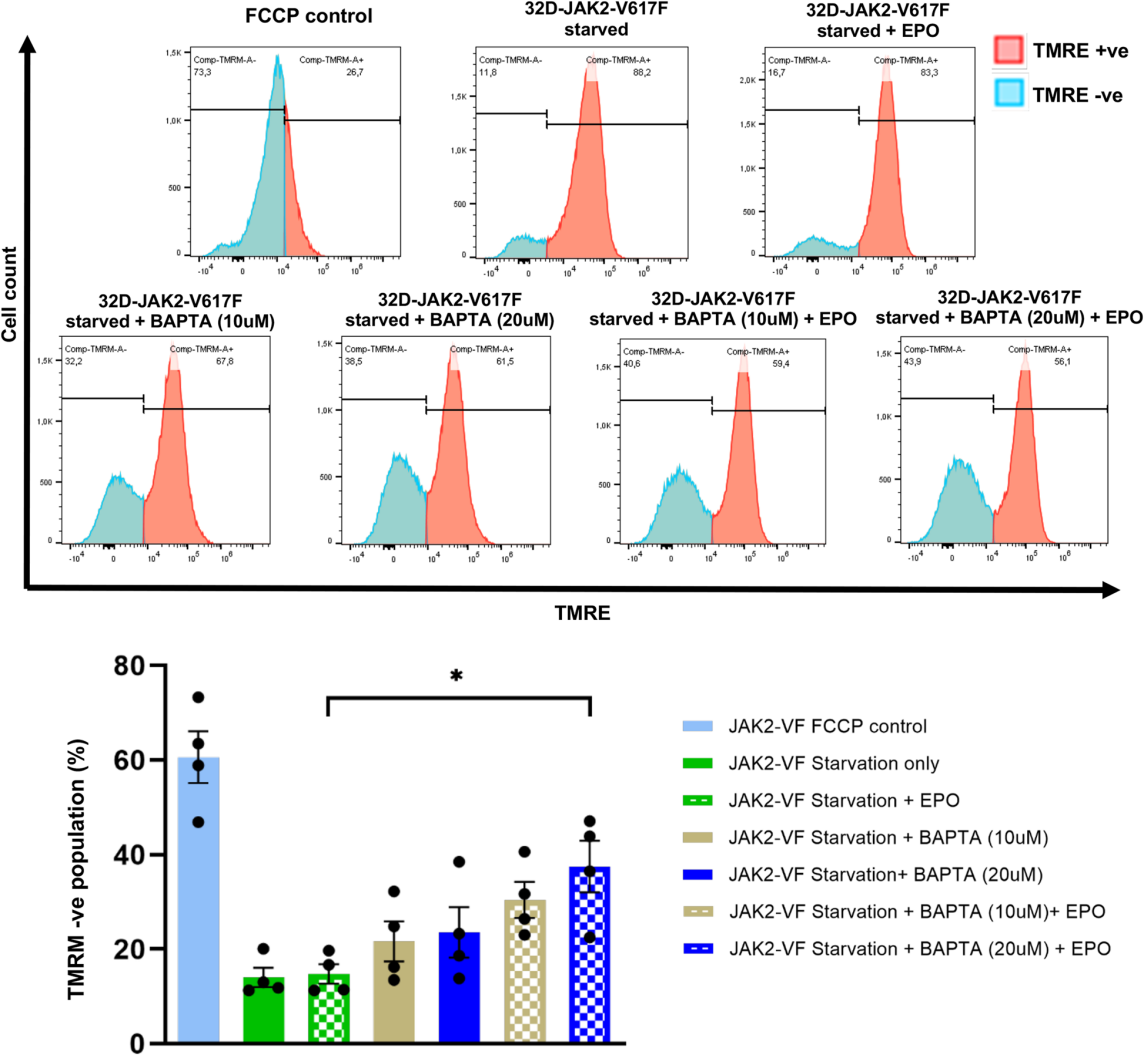
BAPTA treatment induces mitochondrial membrane potential reduction in 32D-JAK2-V617F Cells. (**A**) Representative flow cytometric histogram plots from 4 independent experiments showing the effect of starvation, EPO stimulation and an inhibitory effect of BAPTA on mitochondrial membrane potential in 32D-JAK2-V617F cells. Cells were treated with BAPTA, stained with TMRE and fluorescence was measured by flow cytometry. Data represents mean ± SEM from 4 independent experiments. Statistical analysis by one-way ANOVA by Tukey’s multiple comparison test. * p ≤ 0.05

Mitochondrial depolarization represents a pivotal early event in the initiation of apoptotic cell death. To explore whether BAPTA-induced apoptosis in 32D-JAK2-V617F cells was mediated through mitochondrial death receptors, we conducted an experiment assessing the impact of BAPTA on mitochondrial membrane potential using flow cytometry to measure tetramethylrhodamine ethyl ester (TMRE) fluorescence intensity. Our results demonstrate that BAPTA-treated cells exhibited a significant reduction in mitochondrial membrane potential upon, attributed to the loss of the electrochemical gradient across the mitochondrial membrane (*p* ≤ *0.05*) (Fig. 8).

Next, we aimed to confirm the involvement of JAK2-V617F in the activation of EpoR/JAK2-dependent signaling pathways, with a particular focus on assessing the modulation of JAK2 and STAT activation. As described previously by others [40–43], 32D-JAK2-V617F cells exhibited constitutive phosphorylation of JAK2 and STAT5 proteins in the absence of EPO stimulation, as illustrated in Supplementary Fig. 3A. Moreover, upon EPO stimulation, the level of p-JAK2 in these cells increased significantly by 11.4-fold compared to JAK2-WT cells (Supplementary Fig. 3B). Conversely, the levels of p-STAT3 protein were similar between the JAK2-V617F cells and WT cells under both conditions (Supplementary Fig. 3B). Notably, when the cells were subjected to starvation, the effect of EPO stimulation was further intensified, resulting in a 1.6-fold change in JAK2-V617F cells. This finding underscores the crucial role of EPO in driving the downstream signaling pathways. Besides, the results highlight the potentiation of EPO-driven signaling pathways by starvation, emphasizing the significance of EPO in regulating these pathways. Further, compared to the un-starved condition, an increase in p-STAT3 was detected in both JAK2-WT cells (1.7-fold) and JAK2-V617F cells (1.5-fold) when cells were EPO stimulated. Additionally, while determining the effect of starvation and EPO stimulation on STAT5 protein levels, a 2.1-fold and 2.2-fold change was seen in JAK2-WT and JAK2-V617F cells, respectively (Supplementary Fig. 3B).

This observation highlights the significance of EPO in facilitating cell growth and regulating various cellular processes in both WT and VF cells through the activation of the JAK/STAT signaling pathway. Nonetheless, irrespective of EPO presence and in accordance with literature, our results confirm that the JAK2-V617F activating mutation possesses the capacity to stimulate cell proliferation by triggering the JAK/STAT signaling pathway.

This view is supported by a kinetics experiment on phospho-proteins beginning at 5 min after EPO stimulation and extending up to 60 min to examine the activation patterns of the JAK/STAT signaling nodes. By analyzing the densitometric data, we discovered that JAK2-V617F cells exhibited enhanced responsiveness to EPO in activation of STAT3 (Supplementary Fig. 3C, upper and lower panel). Thus, an increase in p-STAT3 levels was observed in JAK2-V617F cells, with a peak occurring at 5 min after EPO stimulation, whereas the levels and pattern of p-STAT5 remained similar in both cell lines (Supplementary Fig. 3C). Taken together, signalling dynamics in JAK2-V617F cells upon EPO stimulation reveals high activation levels of JAK2, STAT3, PLCγ-1 and IP3R with early activation of PLCγ-1 and IP3R and a slower time course in inactivation of these proteins. This suggests an exceptional capability of JAK2-VF cells to swiftly recognize EPO and then activate cellular processes, favouring them with a distinct advantage over normal cellular regulation. This remarkable phenomenon may enable mutated cells to respond rapidly to exogenous signals, coordinating intricate signaling networks, accelerating vital physiological responses.

### RNA sequencing revealed significant upregulation of calcium-regulated transcriptional activators and of genes involved in cytokine signaling in 32D-JAK2-V617F/EpoR upon EPO stimulation

**Fig. 9:**
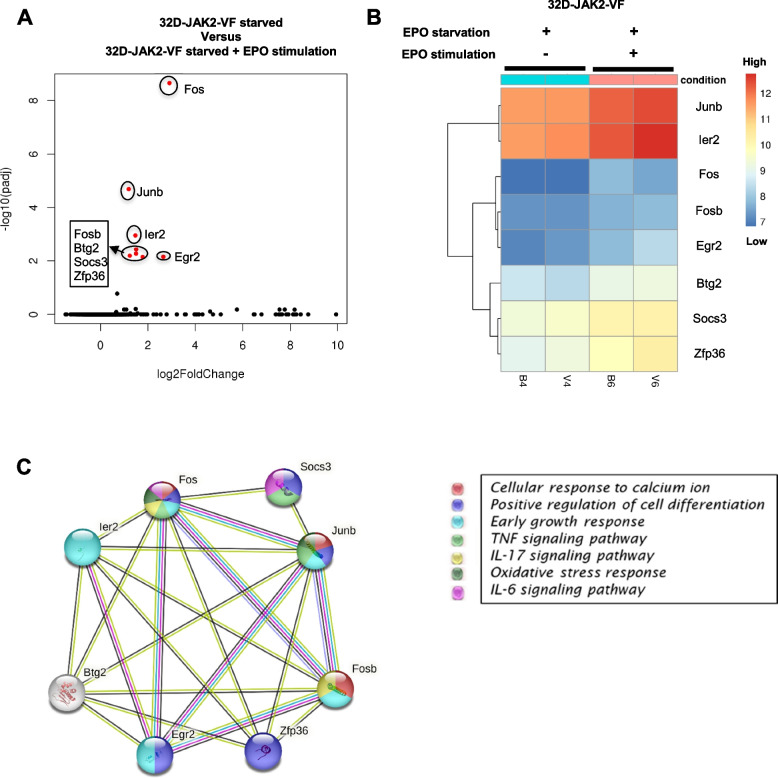
RNA sequencing revealed significant upregulation of calcium-regulated transcriptional activators and of genes involved in cytokine signaling in 32D-JAK2-V617F/EpoR upon EPO stimulation. (**A**) Volcano plot represents overall transcriptional change after EPO treatment. Each data point in the scatter plot represents a gene. The log2 fold change of each gene is represented on the x-axis and the log10 of its adjusted p-value is on the y-axis. Genes with an adjusted p-value less than 0.05 and a log2 fold change greater than 1 are indicated by red dots. These represents significantly up-regulated genes. (Note: There were no downregulated genes observed) (**B**) Bi-clustering heatmap showing the expression profile of the top significantly differentially expressed genes sorted by their adjusted *p*-value by plotting their log2 transformed expression values in samples (**C**) Network interaction image showing the tight interaction between the identified upregulated genes. The number of strings between the connected genes indicate the strength of their interaction. The colours of the spheres designate the biological processes in which the genes participate

To investigate the changes in transcriptome profiles of JAK2-V617F cells upon EPO stimulation**,** RNA sequencing was conducted in 32D-JAK2-V617F cells before and after 25 min EPO treatment. Notably, only eight genes exhibited significant upregulation, while no statistically significant downregulation of any genes was observed. Interestingly, the upregulated genes, such as *Fos*, *Fosb*, and *Junb* function as calcium-regulated transcriptional activators (Fig. 9A) [44, 45]. In addition, they play crucial roles in immune activation cascades [46, 47]. Intriguingly, expression of the early growth response 2 *(Egr2)* gene, responsible for producing immune activation-associated cytokines (IL-6 and TNF), was also upregulated following EPO stimulation (Fig. 9A). Additionally, Zinc Finger Protein 36 *(Zfp36)*, a well-known post-transcriptional regulator of gene expression, demonstrated increased expression (Fig. 9A). Heatmap analysis revealed that *Junb* and *Ier2* were highly expressed compared to other co-expressed genes (Fig. 9B). Subsequently, a gene–gene interaction network of the upregulated genes was constructed (Fig. 9C), demonstrating strong associations among all eight significantly expressed genes. Utilizing string software, various biological and KEGG pathways were identified. *Fos*, *Fosb*, and *Junb* were predicted to be involved in cellular response to calcium ion and IL-17 signaling pathways. Furthermore, *Fos* and *Socs3* were implicated in the IL-6 signaling pathway (Fig. 9C). Moreover, gene ontology analysis of the differentially expressed genes unveiled a prominent signature of pathways associated with calcium ion response, immune activation, cell differentiation regulation, p38/MAPK cascade, and cytokine production (Supplementary Fig. 4). In conclusion, our RNA seq analysis shows that EPO stimulation has a specific impact on RNA up-regulation of a number of calcium-regulated transcriptional activators in 32D JAK2-V617F cells.

## Discussion

In MPNs, genetic alterations in JAK2 and CALR lead to constitutive activation of JAK/STAT signaling pathways, and promote uncontrolled, cytokine-independent proliferation and survival. Constitutive activation of key signaling pathways such as the JAK/STAT pathway and the MEK/ERK pathway are among the main reasons of uncontrolled cell growth and cell survival regulation [48]. In addition, mutated JAK2-V617F cells exhibit hypersensitivity to EPO, wherein multiple mechanisms contribute, including increased JAK2 kinase activity, dysregulation of negative feedback loops, and alterations in EpoR complex composition and function [22, 49].

Calcium signaling, particularly SOCE, regulates cell differentiation, survival, and integrin activation [25, 26]. We have previously shown that in JAK2-V617F mutated cells, VLA4 and LFA1 integrins are over-activated employing Rap1 translocation by a Ca^2+^-dependent activation pathway including CalDAG-GEFI [31]. These results are in line with the here described hypersensitivity in Ca^2+^ flux of SOCE in JAK2-V617F cells. This anticipates that dysregulation of SOCE contributes to altered cellular behavior and may eventually contribute to increased thrombotic propensity [50, 51]. The latter is a clinical hallmark of MPNs and appears to be partly due to hypersensitivity in integrin activation [31]. EPO stimulation was also shown to induce an increase in intracellular Ca^2+^ in human and murine erythroid cells through voltage independent ion channels [52, 53]. Furthermore, EPO is known to induce tyrosine phosphorylation and activation of PLCγ-1, which generates IP3 [54]. IP3 directly binds to its IP3 intracellular receptors (IP3R), releasing calcium from intracellular ER stores to the cytosol and triggering various downstream signaling events mediating fetal erythroid development [55–57]. A study conducted by Qin Tong and colleagues also confirms that EPO stimulation activates PLCγ, leading to the production of IP3. This IP3, in turn, initiates the interaction with TRPC2, a cation channel belonging to the transient receptor potential (TRP) family, which contributes to the opening of ion channels [58]. Besides, in a previously published study by our group, it was shown that PLCγ-1 signaling plays an essential role in EPO-dependent erythropoiesis and erythroid maturation [2]. However, it was not known if intracellular Ca^2+^ homeostasis was affected by JAK2-V617F in the presence or absence of cytokines.

This study elucidates the modulatory effect of EPO and TPO on calcium flux and related signaling pathway activation in JAK2-V617F and CALR mutated cells, demonstrating their consequences on uncontrolled proliferation and survival of these cells. Specifically, we demonstrate that JAK2-V617F cells exhibit heightened sensitivity to EPO, which induces an essential change in the regulatory mechanism of EpoR/JAK2-dependent intracellular Ca^2+^ homeostasis. However, during the quantification of baseline Ca^2+^, we unexpectedly observed lower calcium levels in JAK2-V617F cells in comparison to the WT. While one might have anticipated a chronic elevation in baseline Ca^2+^ levels in JAK2-V617F cells, which has been suggested to negatively impact the growth and proliferation of hematopoietic cells, it is possible that the lower baseline calcium levels observed in JAK2-V617F cells result from a negative regulatory loop [59, 60]. Conversely, TPO stimulation exerts a minor effect on calcium flux in CALR mutated 32D cells. However, in an earlier published study, it was shown that CALR type I megakaryocytes (Mks) demonstrate a marked augmentation in SOCE in contrast to CALR type II mutants [61, 62]. Noteworthy, other previously published research has indicated a suppressive impact on SOCE by CALR overexpression across different cell types [63–65]. In our study, we speculate that the observed resistance of 32D-CALR cells to induce significant calcium flux upon TPO stimulation may be cell-differentiation dependent and thus reflect the biological behavior of undifferentiated 32D myeloid progenitor cells. In addition, there could be divergent pathways regulating the Ca^2+^ flux in CALR-WT versus CALR-mutant cells.

Studies conducted by us and other groups over the past years using the 32D cellular model have delivered biologically meaningful results in a multitude of investigations on JAK2 and FLT3 [17, 66, 67] underlying the appropriateness of this model to study signaling processes. The advantage of using these cells over human leukemic cell lines is the availability of a WT control and the presence of a defined genetic event without any additional cytogenetic aberrations.

Using the 32D cell model, we hereby show that JAK2-V617F triggers an exaggerated response in the phosphorylation of PLCγ-1 and IP3R induced by EPO. Of note, significantly elevated levels of p-PLCγ-1 protein were observed upon EPO stimulation, exhibiting a remarkable 82.9-fold increase compared to unstimulated WT cells. Conversely, CALR mutated cells demonstrated a relatively modest increase in TPO induced p-PLCγ-1 levels, with fold changes of 2.5-fold and 2.1-fold in CALR-ins5 and CALR-del52 cells, respectively, when compared to unstimulated cells. These findings elucidate the enhanced sensitivity of JAK2-V617F mutated cells to cytokine-induced effects. Additionally, our findings indicate that the pharmacologic inhibition of PLCγ-1 or chelating Ca^2+^ using BAPTA, suppresses cellular proliferation and triggers apoptosis in 32D-JAK2-V617F-positive cells by disruption of mitochondrial membrane potential. Although our study primarily examines the effects observed in JAK2-V617F positive cells, we also evaluated the impact on JAK2-WT cells (data not shown). The observed fold increase in apoptosis in JAK2-VF cells was 3.3, whereas in JAK2-WT cells, it was approximately 1.5 (data not shown), indicating a differential response between JAK2-V617F positive and WT cells. Consequently, our research underscores the dual effect of targeting the PLCγ-1 pathway, which not only attenuates cell proliferation but also facilitates the elimination of JAK2-V617F positive cells by promoting apoptotic cell death.

**Fig. 10:**
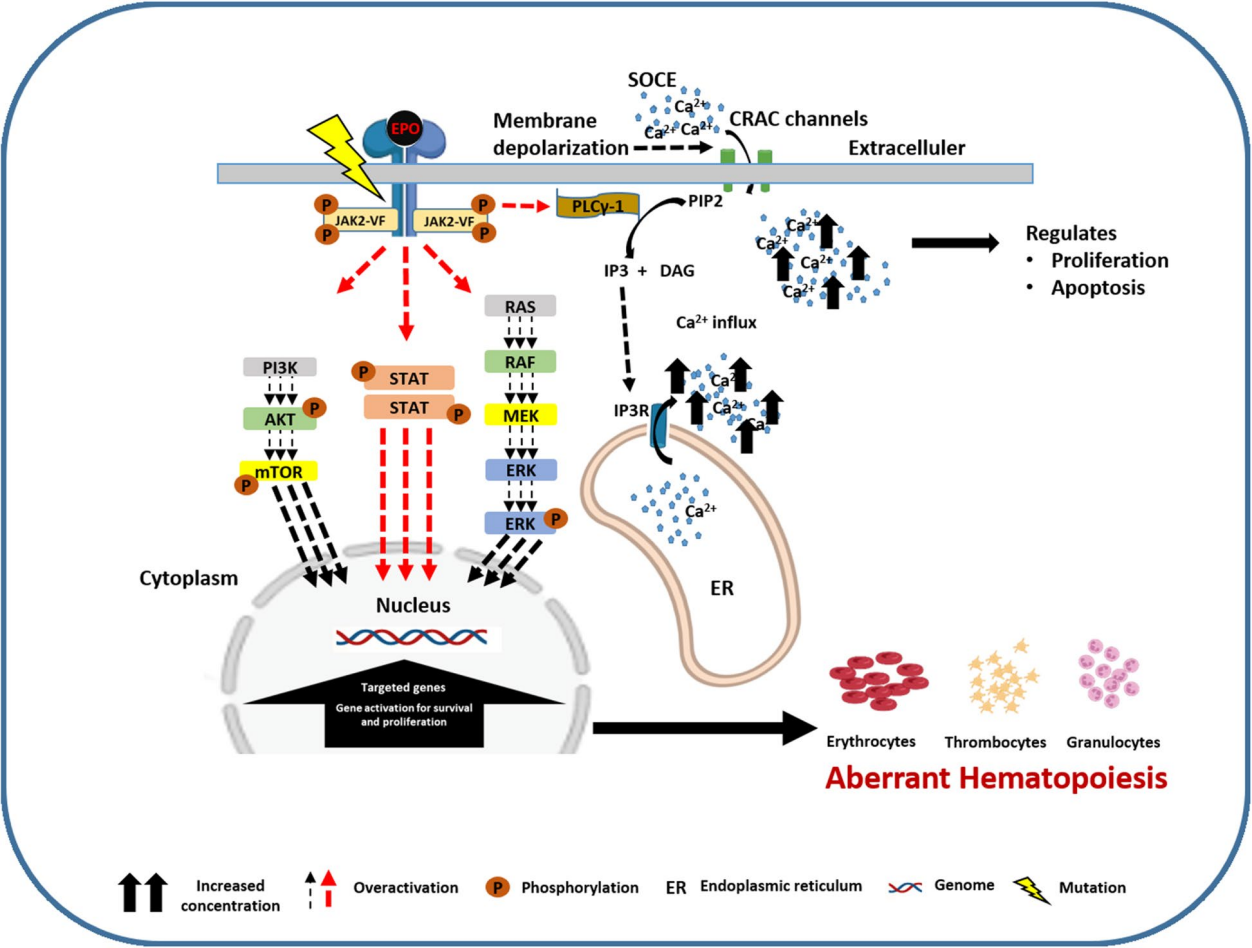
Schematic representation of SOCE activation in 32D-JAK2-V617F cells upon EPO stimulation. The JAK2-V617F mutation induces constitutive activation of the JAK2 kinases. Consequently, multiple tyrosine residues on the EPO receptor undergo phosphorylation. Moreover, JAK2 kinases then phosphorylate signal transducer and activator of transcription (STAT) molecules, facilitating their translocation to the nucleus, where they induce gene transcription. Concurrently, phospholipase C gamma-1 (PLCγ-1) is overactivated, which subsequently generates inositol trisphosphate (IP3). IP3 binds directly to its intracellular IP3 receptors (IP3R), resulting in the release of calcium from the endoplasmic reticulum (ER) stores into the cytosol. Subsequent replenishment of intracellular calcium stores occurs through store-operated calcium entry (SOCE) mediated by transmembrane proteins located on the ER and plasma membrane

Investigating the involvement of JAK2/EpoR downstream signaling in JAK2-V617F mutation expressing cells [22, 68, 69], our results confirm that expression of JAK2 mutation results in a higher activation and an early emergence of the key components (i.e., p-JAK2 and p-STAT3) involved in regulating the cellular proliferation and cell survival in response to EPO [70]. Besides, a delay in the inactivation kinetics of p-PLCγ-1 and of p-STAT3 was also observed. The delay in the inactivation kinetics of activated proteins could derive from a combination of post-translational modifications, feedback loops, cellular compartmentalization, and protein–protein interactions, highlighting the complexity of regulatory mechanisms governing protein activity and signaling dynamics [71–73]. However, for JAK/STAT signaling, various negative regulators have been reported, which include SOCS proteins, tyrosine phosphatases as PTPN1/2 and SHP1/2 and CBL [74]. Our findings are consistent with previous studies that have shown JAK2-V617F mutation-induced EPO hypersensitivity in primary cells from patients. Together, our results are summarized in the scheme presented in Fig. 10.

Moreover, during the examination of global alterations in the transcriptome profile upon EPO stimulation, we observed that EPO stimulation significantly upregulated only 8 genes (i.e. *Junb, Ier2, Fos, Fosb*, *Egr2, Btg2, Socs3, Zfp36*) in 32D-JAK2-V617F cells without any observed downregulation of genes. *Fos*, *Fosb*, and *Junb* are members of the activator protein-1 (AP-1) transcription factor family [47, 75] and are known as “early-response” proteins to stimuli [76, 77]. They play an important role in various cellular processes, including calcium signaling [44, 45]. In an in vivo study in a mouse model, inactivation of *Junb* led to embryonic lethality between embryonic days 6 and 8 [78]. Further, another study suggested *Junb* as an important regulator of erythroid differentiation [79]. Moreover, upon activation, JUNB can interact with FOS or other Jun family members to form active AP-1 complexes [46]. These complexes then modulate the transcription of target genes involved in calcium signaling, neuronal function, and other cellular processes. A gene expression study in a PV patient with JAK2-V617F mutation and healthy donors revealed upregulation of *JUNB* in PV. Further, using Ba/F3-EpoR cell lines and primary human erythroblast cultures, the authors discovered that the transcription factor JunB was activated after the addition of erythropoietin and that the mutated JAK2-V617F gene consistently increased the expression of JunB protein [80]. Additionally, when *Junb* was suppressed, it not only inhibited the growth of Ba/F3 cells by inducing apoptosis but also reduced the clonogenic and proliferative abilities of human erythroid progenitors. Thus, observations from this study indicate that the augmented flow of calcium ions triggered by EPO stimulation synergistically interacts with the persistently elevated levels of Ca^2+^-regulated transcriptional activators in JAK2-V617F cells, promoting the upregulation of genes associated with cellular growth and the induction of resistance against apoptosis.

Upon EPO stimulation, the gene expression of *Zfp36* was also found to be increased in our study. Increased ZFP36 activity following EPO stimulation can have important implications for the regulation of gene expression and cellular responses. Studies have shown that ZFP36 regulates the mRNA stability of several cytokines and chemokines such as TNFα (Tumor Necrosis Factor alpha), IL-3, GM-CSF (Granulocyte–Macrophage Colony-Stimulating Factor), and CXCL2 (C-X-C Motif Chemokine Ligand 2) [81, 82]. ZFP36 in MPN patients, particularly in those with ET and PMF is believed to contribute to the dysregulation of cytokine production and inflammation. This dysregulation may contribute to disease progression, symptom burden, and potentially influence therapeutic responses. Additionally, consistent with our results, other studies have shown that EPO stimulation of JAK2-V617F cells can result in a significant increase in Ier2 and SOCS expression [83, 84]. The upregulation of *Ier2* is believed to be mediated by the JAK2/STAT5 signaling pathway, which is activated upon EPO binding to its receptor. The exact role of increased *Ier2* expression in JAK2-V617F cells after EPO stimulation is not fully understood yet. However, it is suggested that *Ier2* may contribute to the abnormal proliferation and survival of hematopoietic cells observed in MPNs. SOCS3 is a negative regulator of JAK/STAT signaling pathways. EPO stimulation of JAK2-V617F cells has also shown to induce the upregulation of SOCS3 [85]. This upregulation is part of a negative feedback loop to regulate JAK/STAT signaling and prevent excessive activation.

## Conclusion

This report underscores a strong difference between activating JAK2 and CALR mutations on store-operated calcium entry induced by cytokine stimulation with EPO and TPO. Here, we present novel data indicating that the JAK2-V617F mutation brings about a crucial alteration in the regulatory mechanism of EpoR/JAK2-dependent intracellular calcium balance upon EPO stimulation. This alteration affects the levels of calcium both in its baseline state and in EPO-induced SOCE, as well as cytokine-dependent PLCγ-1 signaling pathways. Notably, inhibition of calcium regulatory pathways results in suppression of cellular growth and induction of apoptosis. These findings highlight the important role of calcium flux in the homeostasis of JAK2-V617F positive cells.

### Supplementary Information

**Additional file 1.**

**Additional file 2: Supplementary figure 1**: (A) Experimental design to measure Calcium flux. AUC, area under the curve; SOCE, Store-operated calcium entry; F0, Baseline fluorescence; ER, endoplasmic reticulum

**Additional file 3:** **Supplementary figure 2**: Effects of EPO starvation with and without EPO stimulation on induction of apoptosis in 32D-JAK2-WT and 32D-JAK2-V617F cells. Representative flow cytometric dot plots showing the effect of starvation and stimulation of EPO on early (right lower quadrant) and late (right upper quadrant) apoptosis in 32D-JAK2-WT/EpoR and 32D-JAK2-V617F/EpoR cells. Data represents mean ± SEM from 2 independent experiments. Statistical analysis by one-way ANOVA by Tukey’s multiple comparison test.

**Additional file 4: Supplementary figure 3**: Activating JAK2-V617F mutation induces phosphorylation of JAK/STAT signaling pathways. (A) Western blot analysis of targeted proteins under steady state conditions in 32D-JAK2-WT and 32D-JAK2-V617F cells. Numbers represent values normalized to the respective total protein. (B) Western blot analysis of targeted proteins after 25 min of EPO (5 IU/ml) stimulation following 16hr with or without EPO starvation in 32D-JAK2-WT and 32D-JAK2-V617F cells. Numbers represent values normalized to the respective total protein. (C) Western blot analysis of targeted proteins at various time points after EPO (5 IU/ml) stimulation in 32D-JAK2-WT/EpoR and 32D-JAK2-V617F/EpoR cells. Bar diagrams, represent densitometric analysis of western blots, normalized to GAPDH and expressed as a ratio of phospho and total protein.

**Additional file 5: Supplementary figure 4:** Gene ontology enrichment analysis of significantly differentially expressed genes. (A) Gene ontology term enrichment of EPO stimulated 32D-JAK2-V617F cells, which are significantly enriched with an adjusted P-value less than 0.05 in the differentially expressed gene sets. Statistical analysis performed using Fisher exact test. Data represents mean from 2 independent experiments.

**Additional file 6.**

## Data Availability

All datasets produced or analysed in the present study are presented in the article. The associated Source Data or Supplementary Information files can be obtained from the corresponding author upon a reasonable request.

## Abbreviations

SOCE: Store-operated calcium entry
CRAC: Calcium release-activated calcium
AUC: Area under the curve
ER: Endoplasmic reticulum
EPO: Erythropoietin
EpoR: Erythropoietin receptor
TPO: Thrombopoietin
mTPO: Mouse thrombopoietin
TpoR: Thrombopoietin receptor
MPN: Myeloproliferative neoplasms
CALR: Calreticulin
JAK2: Janus kinase 2
PLCγ-1: Phospholipase Cγ1
JAK2: Janus kinase 2
STAT: Signal transducer and activator of transcription
PI3K: Phosphatidylinositol 3-kinase
Akt: Protein Kinase B
ERK: Extracellular-signal-regulated kinase
MAPK: Mitogen-activated protein kinase
FCCP: Carbonyl cyanide-p-trifluoromethoxyphenylhydrazone
Mks: Megakaryocytes
TNFα: Tumor Necrosis Factor alpha
GM-CSF: Granulocyte–Macrophage Colony-Stimulating Factor
CXCL2: C-X-C Motif Chemokine Ligand 2
IL-3: Interleukin 3
