# Supplementary material for: Activating mutations in JAK2 and CALR differentially affect intracellular calcium flux in store operated calcium entry
Source: Cell Commun Signal. 2024 Mar 21;22:186. doi: 10.1186/s12964-024-01530-z (PMC10956330; doi:10.1186/s12964-024-01530-z)
Supplement: Supplementary file 1 — Additional file 1. [file 12964_2024_1530_MOESM1_ESM.docx]

| **S.No** | **Antibody** | **Supplier** | **Catalog no.** | **Dilution** |
| --- | --- | --- | --- | --- |
| 1 | p-JAK2 (Tyr1007/1008) | Cell Signaling | #3771 | 1:1000 |
| 2 | t-JAK2 | Cell Signaling | # 3230 | 1:1000 |
| 3 | p-PLCγ1 (Tyr783) | Cell Signaling | #2821 | 1:1000 |
| 4 | t-PLCγ1 | Cell Signaling | #5690S | 1:1000 |
| 5 | p-IP3R (Ser1756) | Cell Signaling | #3760 | 1:1000 |
| 6 | t-IP3R | Cell Signaling | #8568 | 1:1000 |
| 7 | p-STAT3 (Ser727) | Cell Signaling | #9134 | 1:1000 |
| 8 | t-STAT3 | Cell Signaling | #9139 | 1:1000 |
| 9 | p- STAT5A/B (Tyr694/699) | Millipore | #05-495 | 1:1000 |
| 10 | t- STAT5A | Santa Cruz | #sc-271542 | 1:500 |
| 11 | GAPDH | Meridian Life Science | #H86504M | 1:5000 |
| 12 | Vinculin | Cell Signaling | #13901 | 1:1000 |
| 13 | Sytox-Blue | Invitrogen | # S34857 | 1:300 |
| 14 | Alexa Fluor® 647 Annexin V | Biolegend | #640912 | 4:200 |
| 15 | Anti-mouse IgGκ, HRP | Santa Cruz | #sc-516102 | 1:2000 |
| 16 | Anti-rabbit IgG, HRP | Cell Signaling | #7074 | 1:2000 |

**Antibodies**
