# Supplementary figures and images for: Activating mutations in JAK2 and CALR differentially affect intracellular calcium flux in store operated calcium entry
Source: Cell Commun Signal. 2024 Mar 21;22:186. doi: 10.1186/s12964-024-01530-z (PMC10956330; doi:10.1186/s12964-024-01530-z)

A

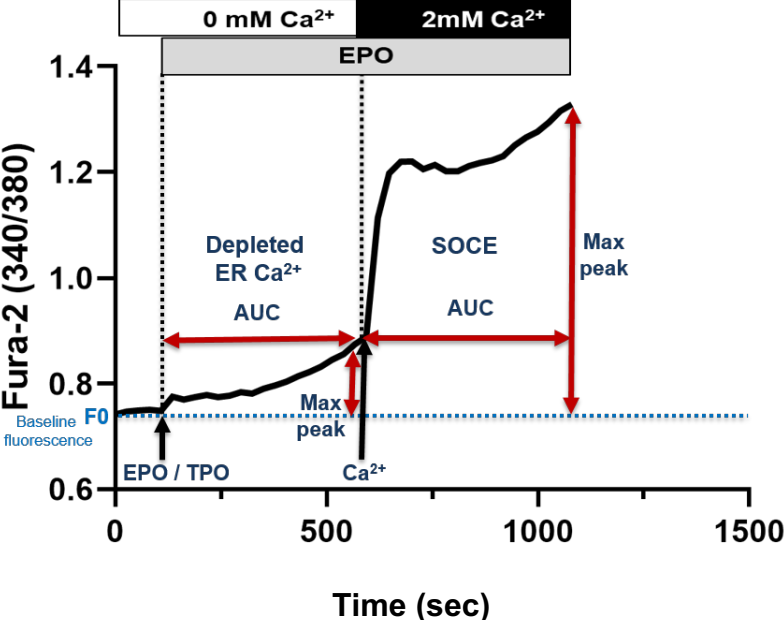

Supplement: Supplementary file 2 — Additional file 2: Supplementary figure 1: (A) Experimental design to measure Calcium flux. AUC, area under the curve; SOCE, Store-operated calcium entry; F0, Baseline fluorescence; ER, endoplasmic reticulum [file 12964_2024_1530_MOESM2_ESM.pdf]

A

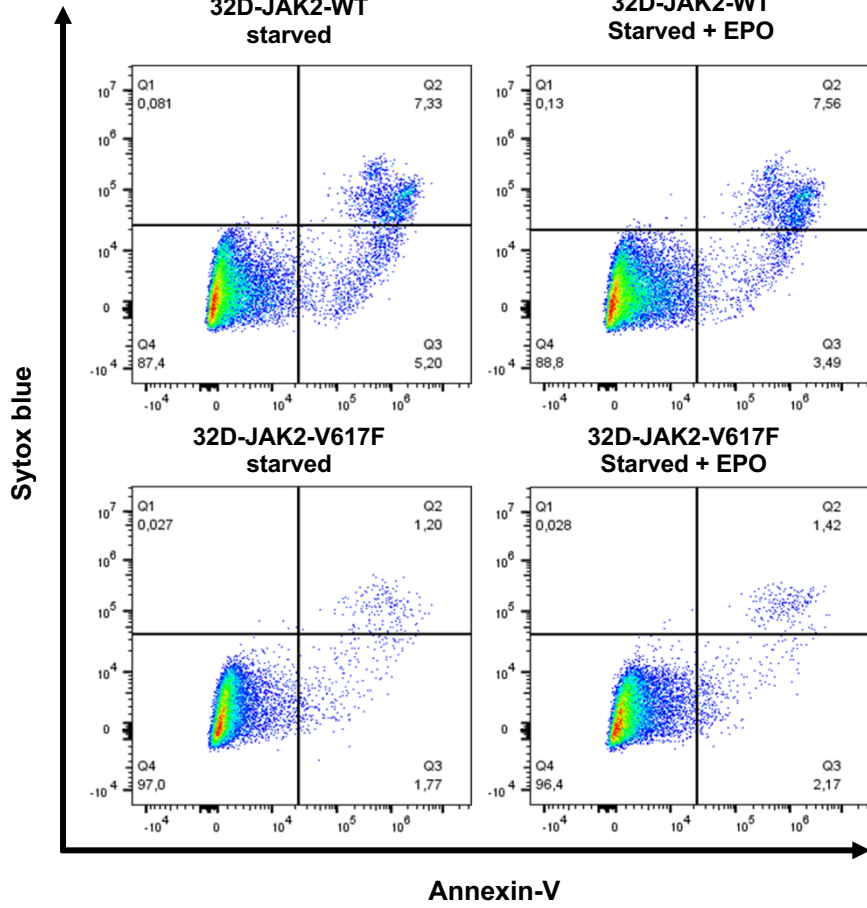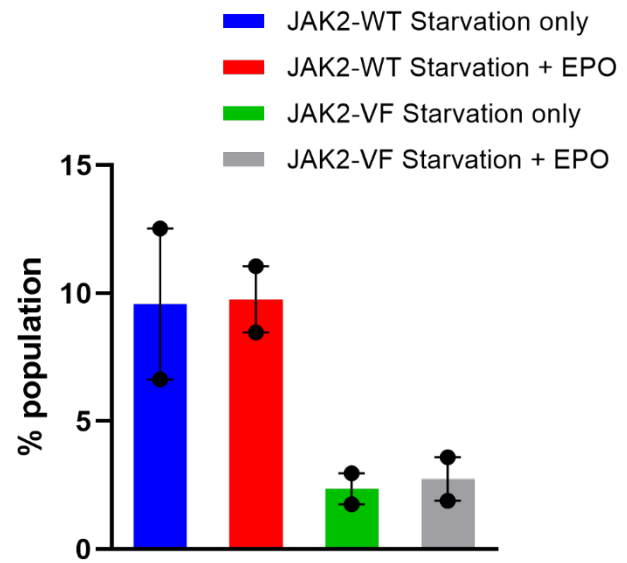

Supplement: Supplementary file 3 — Additional file 3: Supplementary figure 2: Effects of EPO starvation with and without EPO stimulation on induction of apoptosis in 32D-JAK2-WT and 32D-JAK2-V617F cells. Representative flow cytometric dot plots showing the effect of starvation and stimulation of EPO on early (right lower quadrant) and late (right upper quadrant) apoptosis in 32D-JAK2-WT/EpoR and 32D-JAK2-V617F/EpoR cells. Data represents mean ± SEM from 2 independent experiments. Statistical analysis by one-way ANOVA by Tukey’s multiple comparison test. [file 12964_2024_1530_MOESM3_ESM.pdf]

**A**

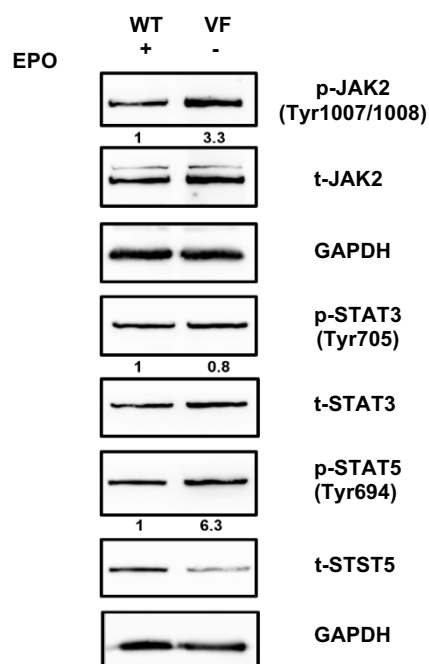

**B**

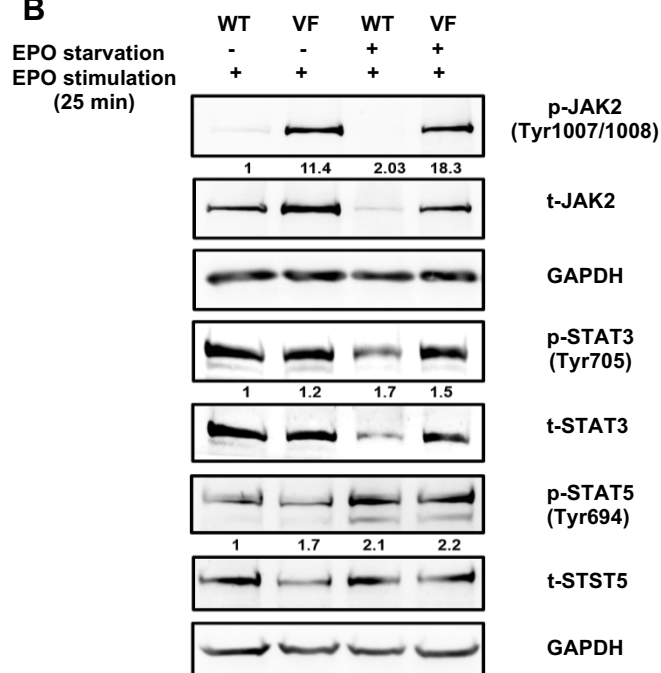

**C**

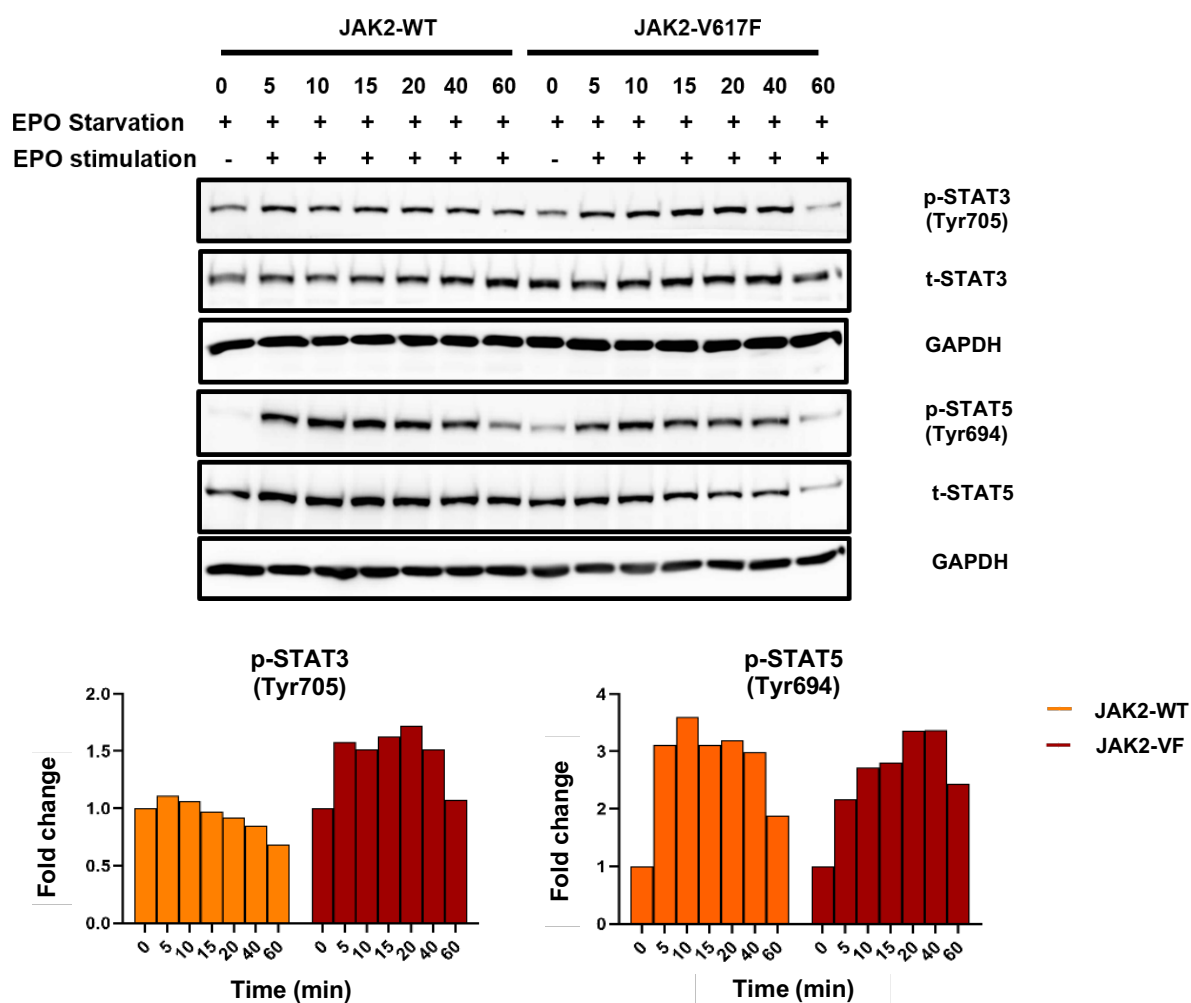

Supplement: Supplementary file 4 — Additional file 4: Supplementary figure 3: Activating JAK2-V617F mutation induces phosphorylation of JAK/STAT signaling pathways. (A) Western blot analysis of targeted proteins under steady state conditions in 32D-JAK2-WT and 32D-JAK2-V617F cells. Numbers represent values normalized to the respective total protein. (B) Western blot analysis of targeted proteins after 25 min of EPO (5 IU/ml) stimulation following 16hr with or without EPO starvation in 32D-JAK2-WT and 32D-JAK2-V617F cells. Numbers represent values normalized to the respective total protein. (C) Western blot analysis of targeted proteins at various time points after EPO (5 IU/ml) stimulation in 32D-JAK2-WT/EpoR and 32D-JAK2-V617F/EpoR cells. Bar diagrams, represent densitometric analysis of western blots, normalized to GAPDH and expressed as a ratio of phospho and total protein. [file 12964_2024_1530_MOESM4_ESM.pdf]

A

**32D-JAK2-VF starved + EPO stimulation**

GO Terms

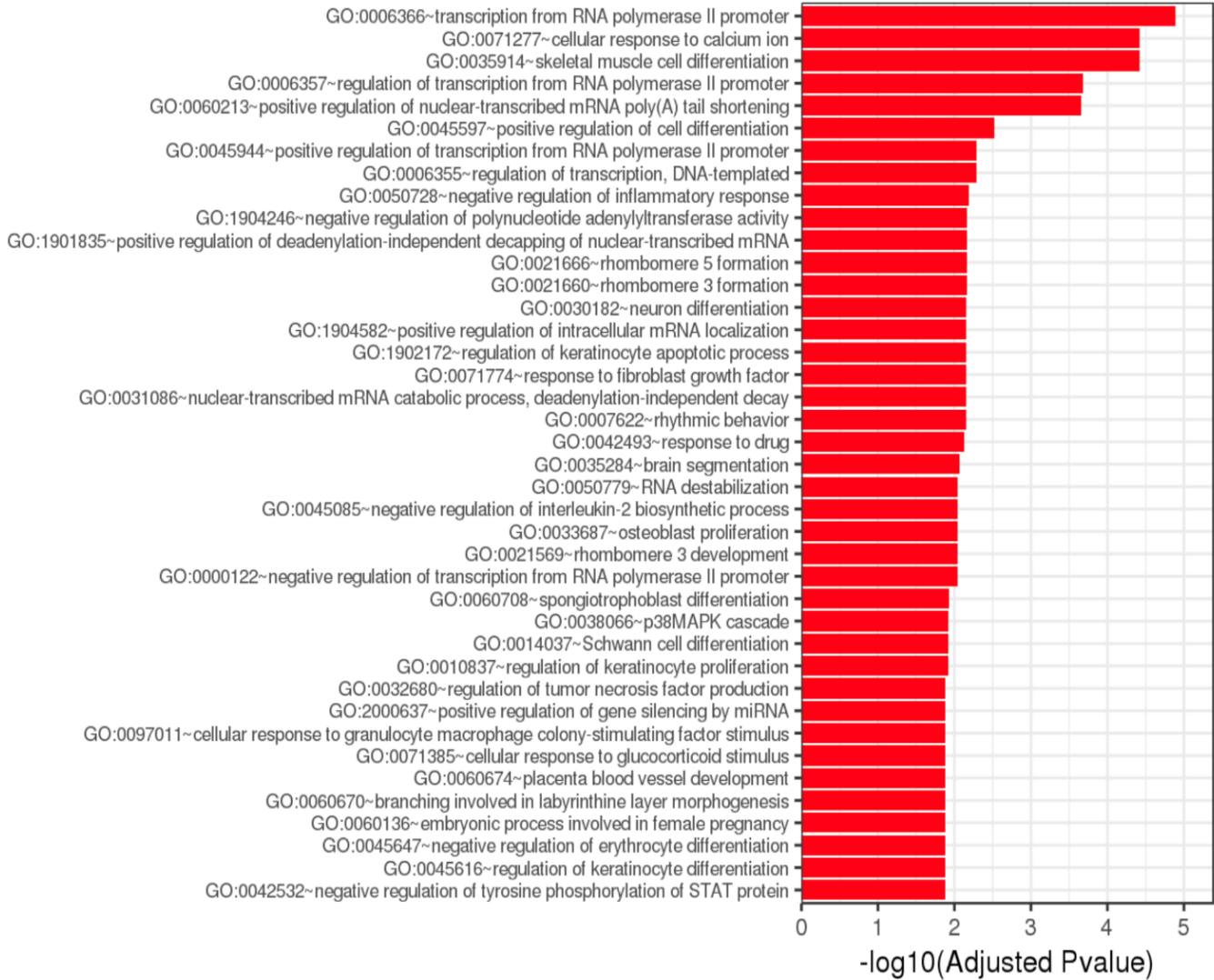

Supplement: Supplementary file 5 — Additional file 5: Supplementary figure 4: Gene ontology enrichment analysis of significantly differentially expressed genes. (A) Gene ontology term enrichment of EPO stimulated 32D-JAK2-V617F cells, which are significantly enriched with an adjusted P-value less than 0.05 in the differentially expressed gene sets. Statistical analysis performed using Fisher exact test. Data represents mean from 2 independent experiments. [file 12964_2024_1530_MOESM5_ESM.pdf]
