# Supplementary material for: Activating mutations in JAK2 and CALR differentially affect intracellular calcium flux in store operated calcium entry
Source: Cell Commun Signal. 2024 Mar 21;22:186. doi: 10.1186/s12964-024-01530-z (PMC10956330; doi:10.1186/s12964-024-01530-z)

# Supplementary file
# (Western blots)

## FIGURE-2

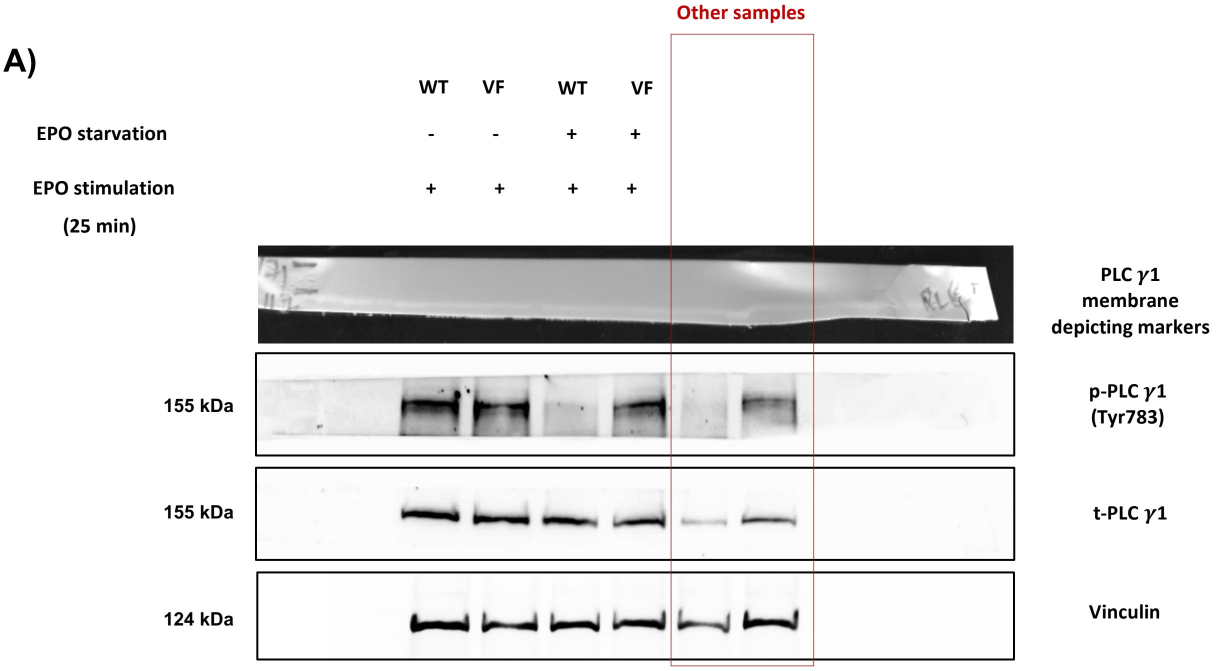


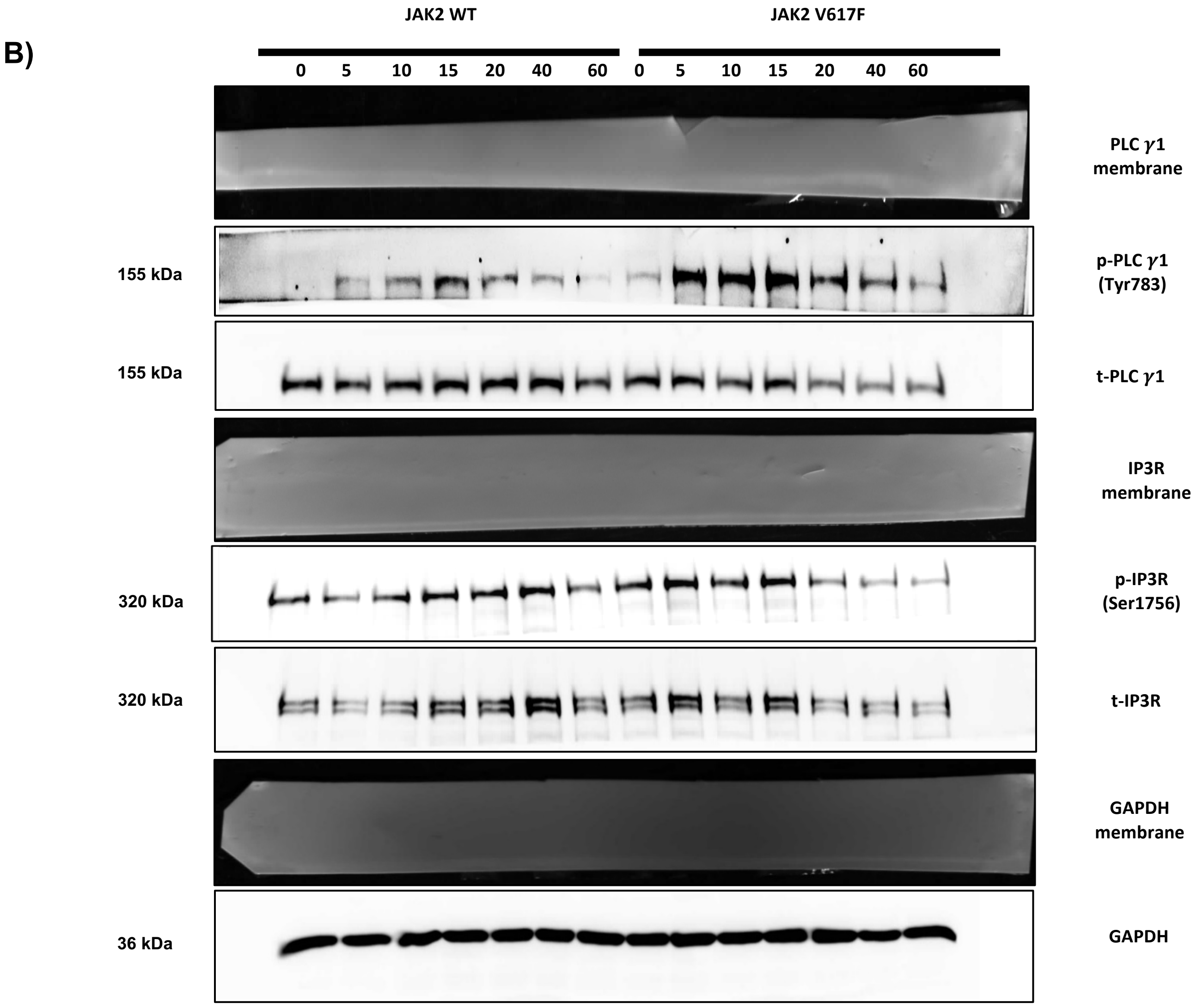

# FIGURE-3

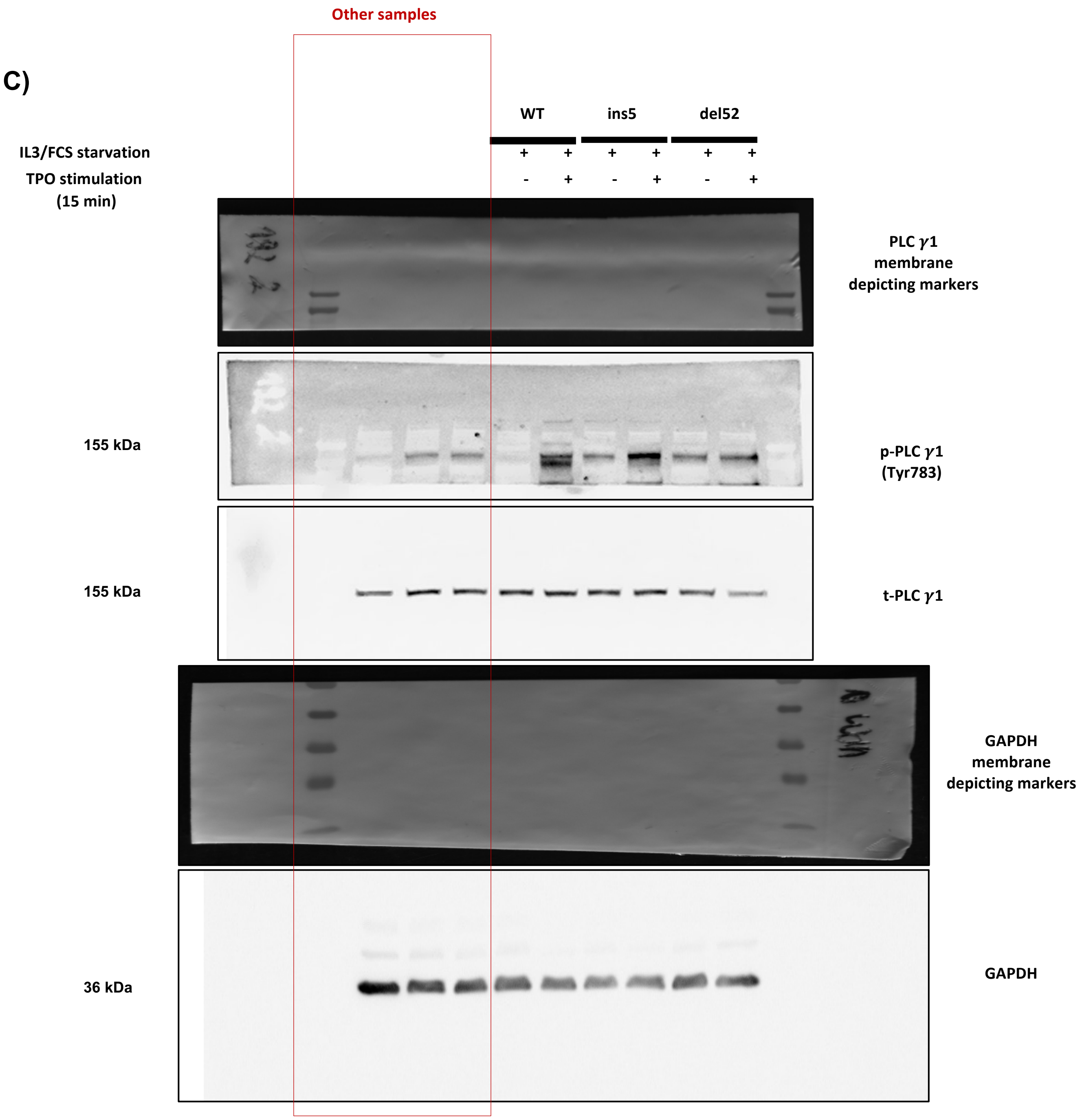
Other samples
C)
WT
ins5
del52
IL3/FCS starvation
+ + + + + +
TPO stimulation
(15 min)
- + - + - +
PLC γ1
membrane
depicting markers
155 kDa
p-PLC γ1
(Tyr783)
155 kDa
t-PLC γ1
GAPDH
membrane
depicting markers
36 kDa
GAPDH

# FIGURE-5

B)

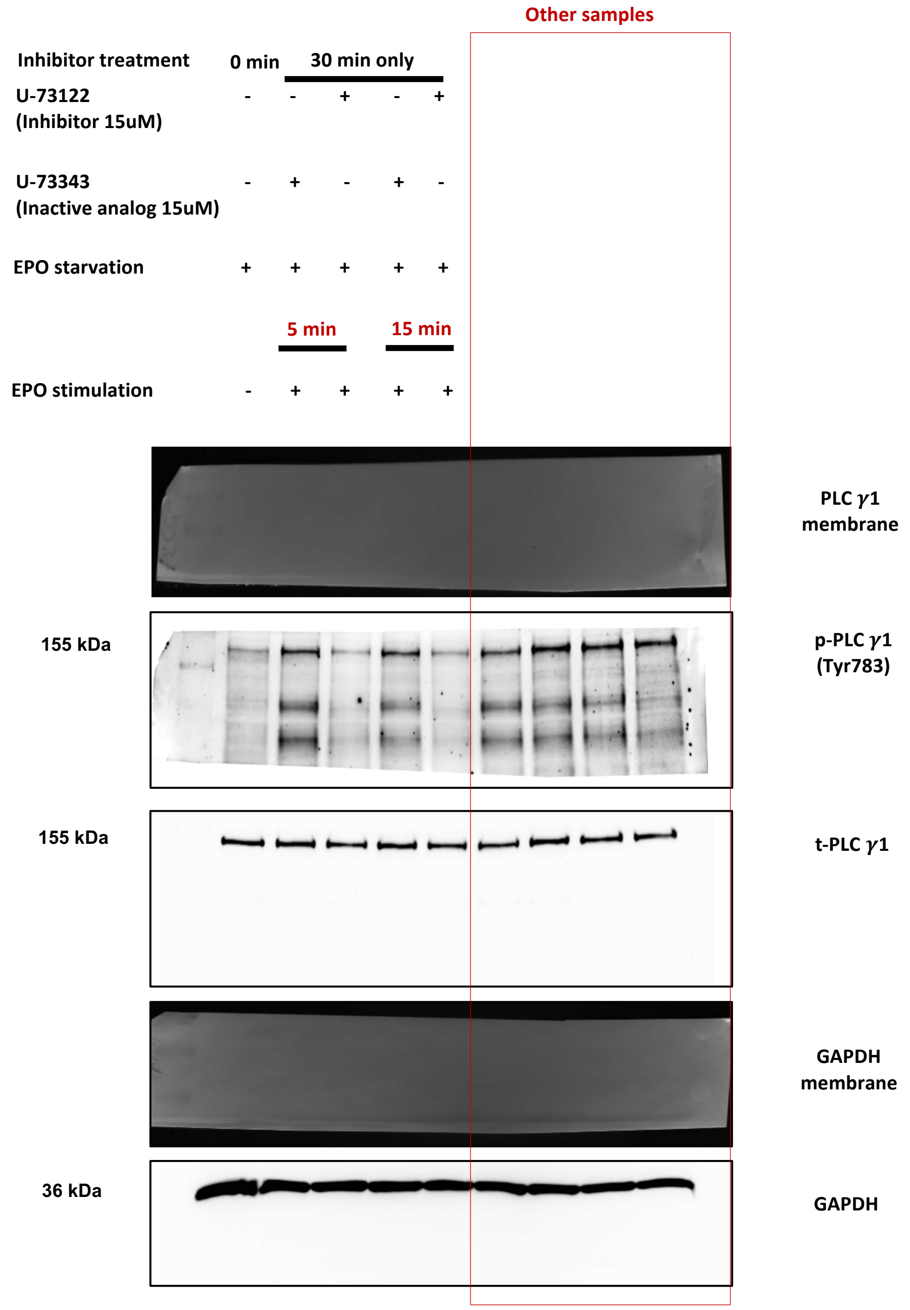
Other samples
Inhibitor treatment
0 min
30 min only
U-73122
(Inhibitor 15uM)
- - + - +
U-73343
(Inactive analog 15uM)
- + - + -
EPO starvation
+ + + + +
5 min
15 min
EPO stimulation
- + + + +
PLC γ1
membrane
155 kDa
p-PLC γ1
(Tyr783)
155 kDa
t-PLC γ1
GAPDH
membrane
36 kDa
GAPDH

# FIGURE-7

## A)

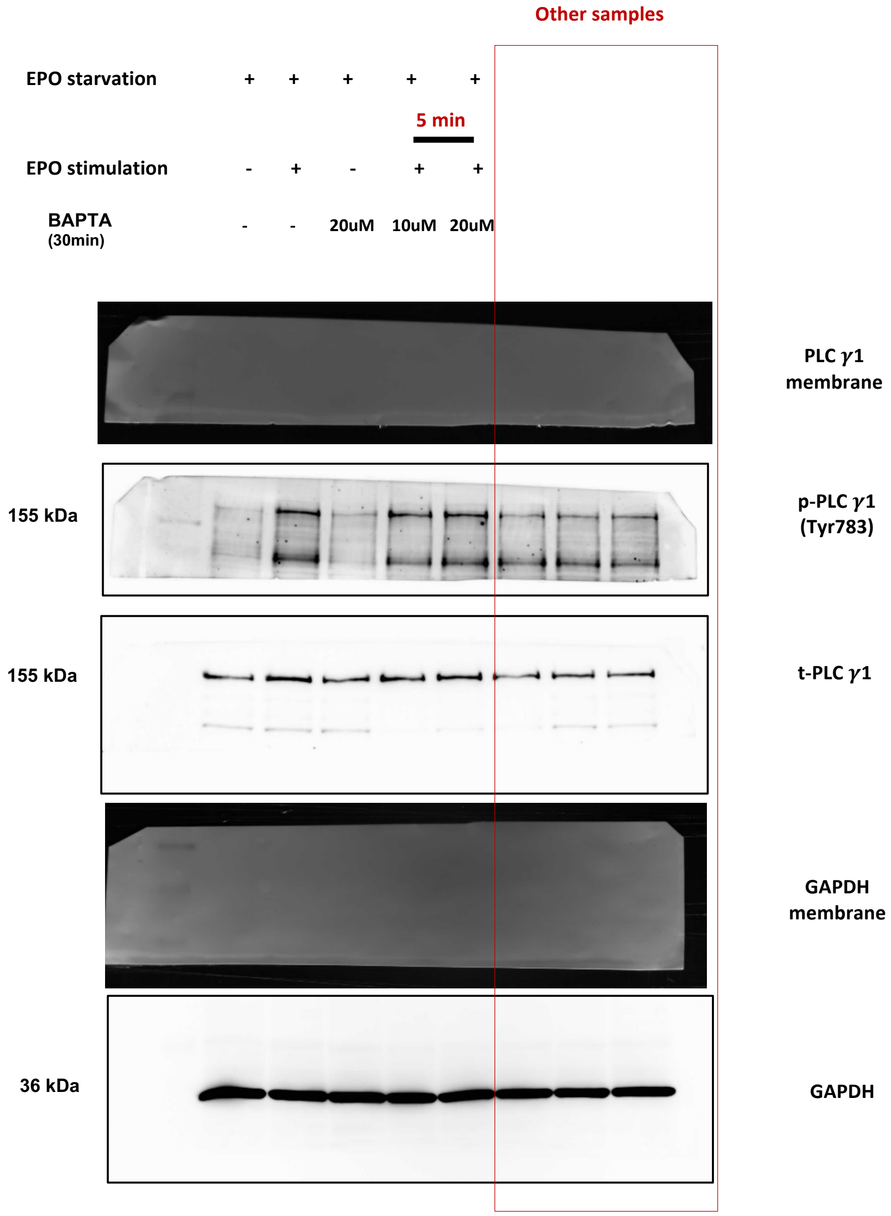
Other samples
EPO starvation + + + + +
5 min
EPO stimulation - + - + +
BAPTA (30min) - - 20uM 10uM 20uM
PLC γ1 membrane
155 kDa
p-PLC γ1 (Tyr783)
155 kDa
t-PLC γ1
GAPDH membrane
36 kDa
GAPDH

# Supplementary figure 3

A)

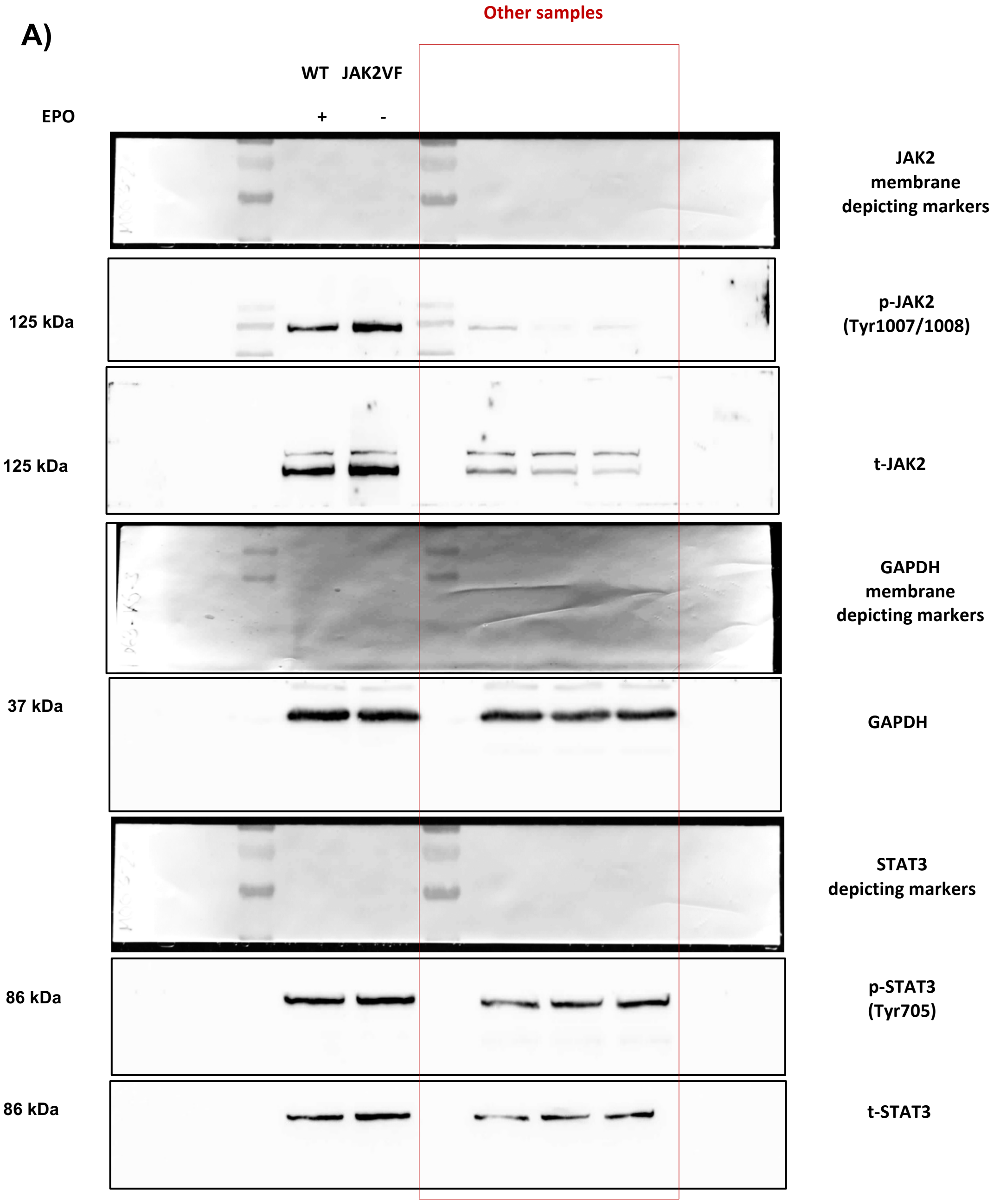

# Supplementary figure 3

A)

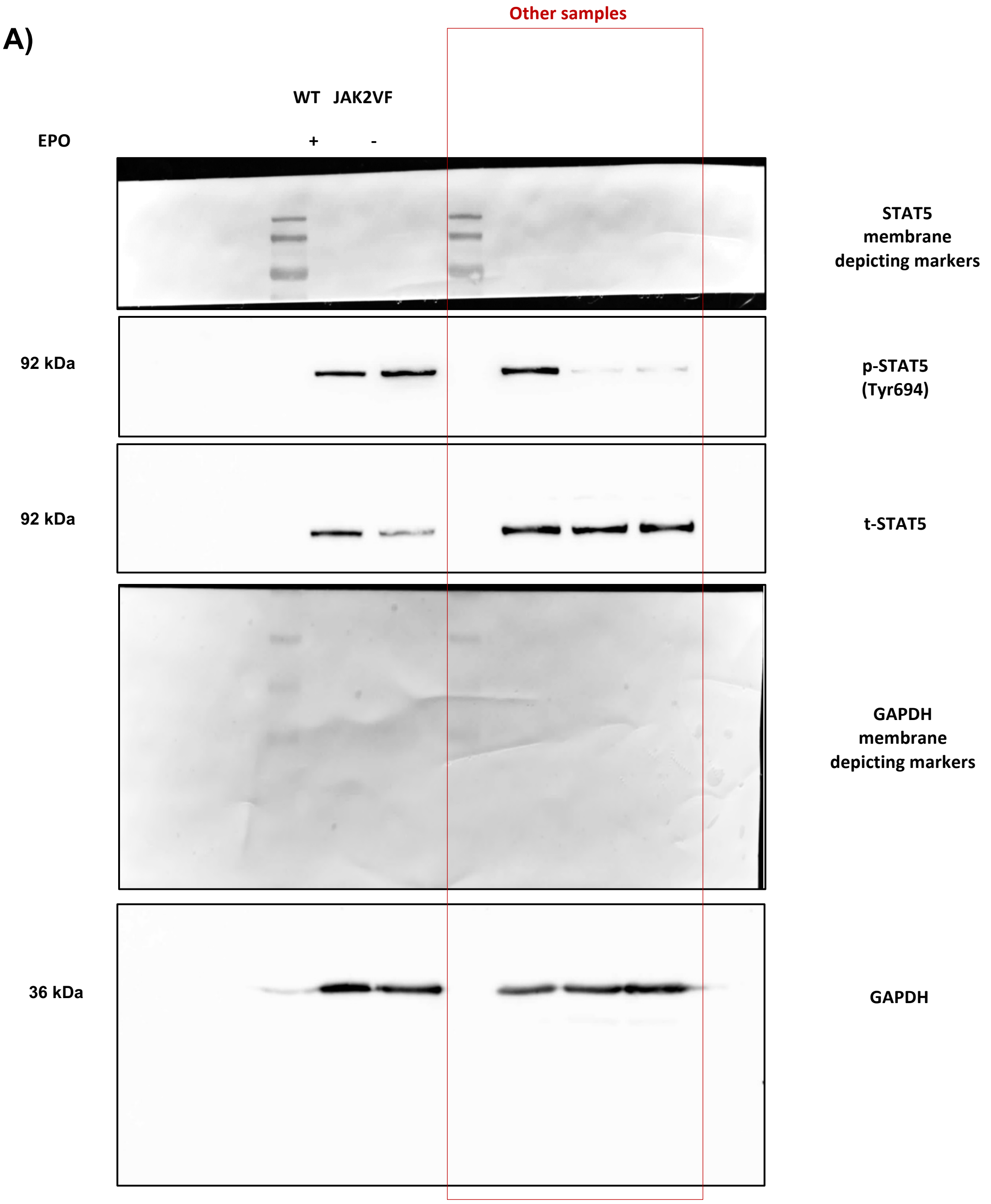

# Supplementary figure 3

B)

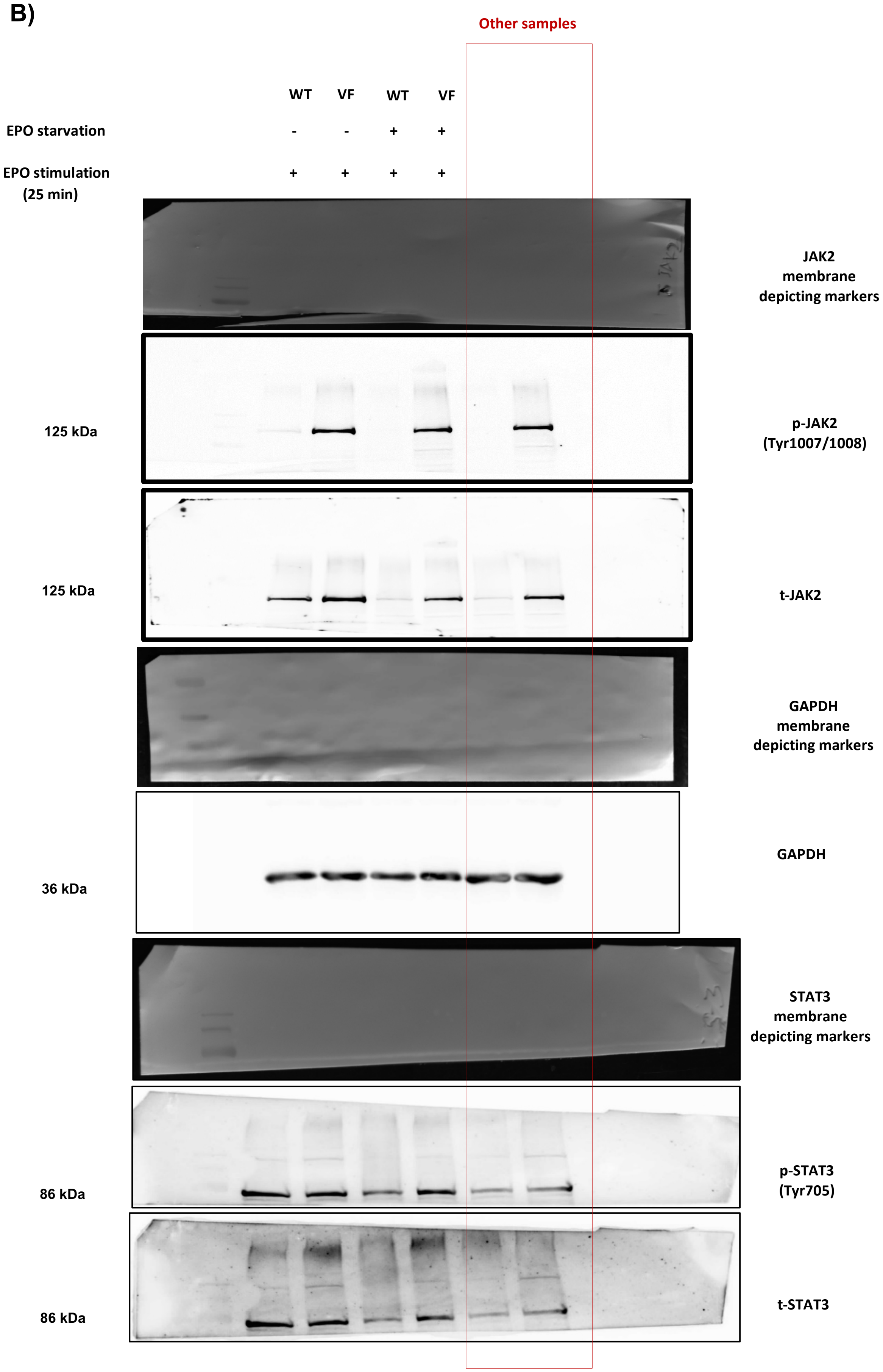

## Supplementary figure 3

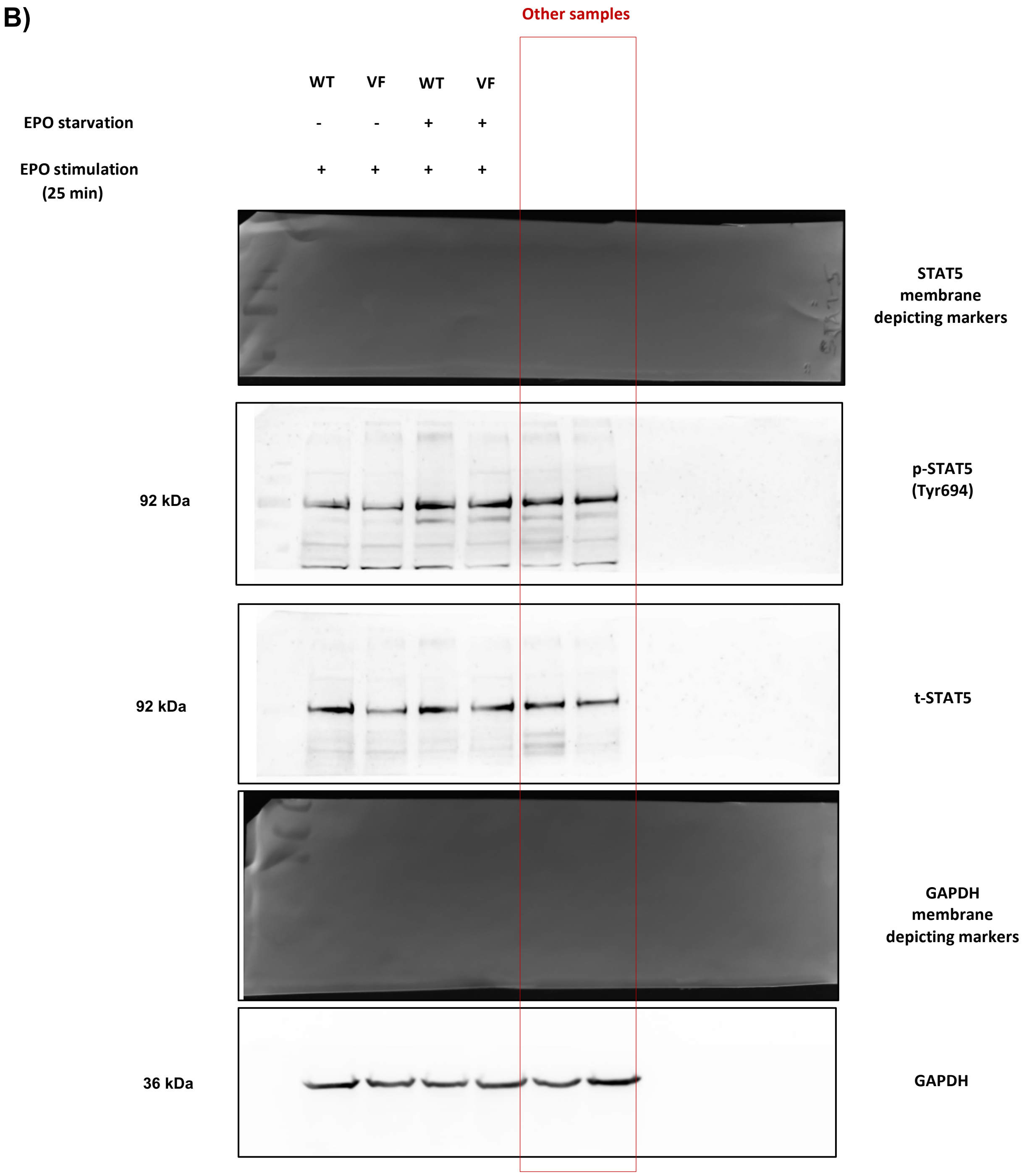

# Supplementary figure 3

C)

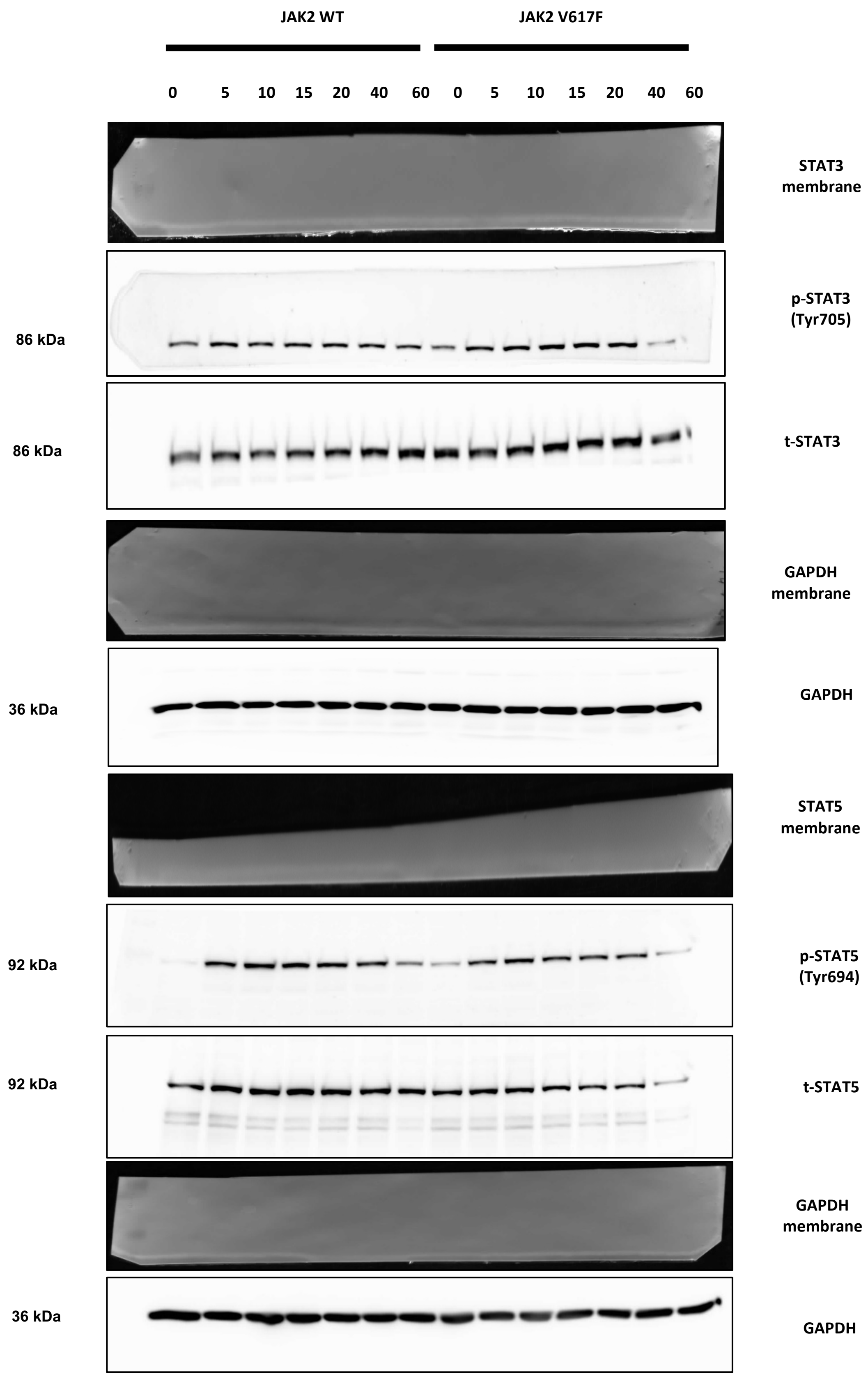

Supplement: Supplementary file 6 — Additional file 6. [file 12964_2024_1530_MOESM6_ESM.pdf]
